# Supplementary material for: Challenges in accessing health care and socio-protection services among children living and working in streets in northwestern Tanzania: A qualitative study
Source: PLOS Glob Public Health. 2023 May 17;3(5):e0001916. doi: 10.1371/journal.pgph.0001916 (PMC10191300; doi:10.1371/journal.pgph.0001916)
Supplement: S1 Data — (ZIP) [file pgph.0001916.s001.zip › Data/FANISI ENGLISH 1.docx]

**FANISI ORGANIZATION**

***Interview between the staff of the Fanisi organization***

**Questioner:** I would like to know the origin of fanisi, when did the Fanisi organization start and what does it deal with?

**Mr.X1:** Fanisi is a non-governmental organization and it was founded in 2019. Fanisi organization originates from Mwanza, Ilemela district.

**Questioner:** What are your particular responsibilities as a social organization here in Mwanza?

**Mr. X1:** In general, Fanisi as an organization is based at the community level and works with the families and society as a whole.

**Miss X2** As Gabriel said, there are many organizations, including the MOSCO organization, which provides health insurance to children living and working on the streets. We decided to go to prevention so that we cut the roots there, and we decided to prevent children from going to the streets so we created our own interventions, we have, Family intervention which focuses on providing therapy sessions for children and parents about the problems they encountered in their lives, Community intervention we provide positive sessions for parents and we also have clubs that we visit every month to educate parents about positive education and upbringing better to prevent child abuse.

School intervention itself is focused on child abuse prevention, providing education against violence, how can children protect themselves, if they experience violence, how should they know where the place to give information is, parents also get organized and make small economical groups to support themselves financially and the goal is for mothers to be able to live and meet the needs of their families in a safe way so that the child does not leave the house and go to the streets. I believe if a parent can raise his child well we will not have many cases of street children the children will be raised in better environment.

**Mr X3:** Fanisi, we are currently implementing two projects, where we have a parenting project, (the Malezi Mothers Project) which includes all the interventions that my colleague has mentioned. This project is implemented in three districts which are; Sengerema District, Nyamagana District and Ilemela District. We also have another project that is being implemented in Sengerema district alone. This project makes the child and the parent realize their basic rights but we also provide education for the parents to realize the rights of the child so as the children to recognize theirs.

***Questioner: What exactly are you doing to help these children who live and work on the street healthily?***

**Miss X2** we at fanisi are not involved with street children at all, we are involved with children in the community. In terms of health. Fanisi provides health assistance to the children, including transportation to get relevant services at the hospital. Fanisi gets support and help from the local donors, the local donors have been of great help in insuring the children get health services.

**Mr. X1:** Fanisi does not have a specific budget for the health issue, and we see that there is a great need for health in the community. We are talking to various people and companies. Also, people are offering to provide insurance for the child’s health though it’s from a smaller extent, in addition to that we encourage parents to form those financial supportive groups among themselves so as to raise and support their children well.

***Questioner: What exactly are you doing to protect children who live and work on the streets and those involved with difficult situations in terms of sexual violence and violent acts?***

**Mr X1:** first of all we have invested in awareness, we as fanisi have seen the importance of guiding the child to get an education on how he can protect himself against dangerous situations, from different people, with this information he will be able to know where to report from, the child will know his rights, the parents and the community will also be brought into awareness.

***Questioner: What methods do you use to educate the children? Do you gather them together or you speak to them individually?***

**Answer 1:** The methods we use to reach three quarters of the children, we find them in institutions. In the Mwanza region, many children are in schools, so we thought it’s the best way to provide them with education, we have started with few schools, 30 schools to be specific, we have started something called a class clubs in schools, which is against child abuse. In each school there are at least 20 members in each club, we work hand in hand with teachers. We have noticed a change, the children now recognize any forms of child abuse, and once they experience they tend to report to the club leaders and the club leaders inform the teachers and the teachers convey the information to us.

**Miss X2** We empower them through guidelines that are developed from other organizations and we decided to develop our own guide here in Tanzania that will guide us in the issues of child abuse.

***Questioner: What methods do you use to ensure that children living in dangerous environments are protected?***

**Answer 1:** we at Fanisi have a procedure of working with the local government leaders, so the family will be recognized by the local Government leaders, especially the Chairman and the Councilor. So, once the chairman brings us the number of families who live in a harsh environment, we usually go find families that need health insurance and incase they experience challenges we usually accompany them to Bugando Hospital, we usually pay the expenses ourselves, but also we as Fanisi don’t isolate ourselves we coordinate with other organizations with the same vision of assisting the children, incase the child is at high risk, we usually send the child to children's shelters such as Forever Angels, Fonelisco.

***Questioner: What are the methods you use to ensure that children living in dangerous environments are protected from sexual and social violence?***

**Mr. X3:** we don't just provide education, but we have a family department and a school department, the family department does a follow-up in the family, it recognizes the risks that the child faces and also finds a solution to the challenges that the child is going through and makes sure the child is safe.

***Questioner: How many children who live in dangerous environments do you help them in getting and enabling them to get health services?***

**Mr. X1:** we have a lot of needy people like 1400 children with health needs who are all vulnerable. And we are not focused much on helping those children, but we are trying to find local donors, organizations that are focused on the health sector to save those children.

***Questioner: in that group of children have you tried to protect them from sexual and social violence, how many females are there?***

***Mr. X3:*** we have had many reports on male children, and these have led to a sub project that is related to cases of sodomy, although there are cases of female children, but they are not as many as the male children.

***Questioner: What aid do you provide as a social organization in helping children living in vulnerable environments to access health services and protect them from sexual and social violence?***

***Mr. X1:*** according to our information, our benefactors have invested a lot in awareness than material things, we have an initiative which is inviting local donors and other organizations as well as stakeholders to conduct a campaign to collect school supplies so that the child does not stay at home due to lack of clothes or notebooks different Individuals volunteer to help them with health issues.

***Miss X2*** there are children who have nutritional problems, we at Fanisi support them as organization, we go to the doctors, they prepare for us what kind of nutrition the child with these problems should have at the right time.

***Questioner: what are the specific obstacles you encounter as an organization when you provide support to children living in dangerous environments?***

***Mr. X1i:*** the mentality of our community, we Tanzanians, the communities believe so much in receiving material things from the organizations, when we organize meetings for educational purposes we get less response, since it doesn’t reach their expectations.

***Questioner: what are the specific challenges you encounter in your activities as a social organization dealing with children living in dangerous environments?***

***Miss X2*** we have very few donors compared to those in need in our communities, so there is great need for our management to find donors so that we can reach our organizational goal.

***Questioner: what are the specific challenges that you encounter in ensuring that children living in dangerous environments get health services and are protected against sexual and social violence?***

***Mr. X1:*** There is no transparency from the community, for example a child is brutalized by a violent child, there is little cooperation from the child's family in identifying the person who brutalized the child, so the incident is blinded to their goal, they know that they are protecting the honor of the family, but we Fanisi realize that the child is affected and can be harmed, if the person responsible is identified, we take the person responsible/perpetrator of the crime in the relevant area and the law continues to act.

***Miss Alloudia:*** we get complaints from stakeholders that many children have been brutalized but doctors do not provide evidence so that the child can get justice, and if the person involved is caught and released, he’s likely to go back to the streets hence he’s likely to commit the same crime, so in the society the children continue getting hurt.

***Questioner: What are the obstacles you encounter as an organization involved in helping children living and working on the streets to ensure they are protected against sexual and social violence.***

***Mr. X1:*** after finding out that a child has experienced violence we use the organization's guidelines and see how we can assist the child into getting back to normal state.that we organize special sessions in schools so that we can help the child to return to his normal state, we have received cases of children who gave up and dropped out of school, we still try to comform more as we can so that we don’t lose any more of them.

***MR. X3:*** We have had the opportunity to get funds from donors where, we rely on donors to complete the goals of the organization.

***Questioner: How do you work with health departments, as well as health care centers to ensure that children who live and work on the streets get health services and are protected against sexual and social violence?***

***Mr.X3:*** we as Fanisi, have reached other organizations that are in the health sector to achieve the goal of protecting the child.

***Mr. X1:*** we work with CUHAS, the social welfare and community development officers found in every hospital, Buzuruga Hospital, BEIRA, and clinics, all these work with us in educational provision for the parents and guardians.

***Questioner: How do you work with the surrounding community to ensure that children who live and work in the street get health services to protect them from sexual and social violence?***

***Mr. X1:*** in the community we work with guardians at the level of wards where we have created clubs, each club has 60 members who meet every month for training. We are making efforts to reach local, although we are reaching a few, we focus more on community gatherings. Another approach which is less costly due to our shortage of funds, we plan to meet mothers at clinics to educate them on how they will be able to create positive relationships with their children.

***Questioner: What are the opportunities available to help children living and working on the streets in accessing health services and protecting them from gender and social violence?***

***Mr. X1:*** until now we can achieve our activities through support from the government social welfare and development sector, local government executives and leaders,through them we had an easy and fast access to meet the community.

***Questioner: What methods do you use to find or get opportunities?***

***Mr. X3:*** currently we have a board in the organization that is responsible for finding Donors, grants.

***Questioner: what methods do you use to ensure that the opportunities that arise reach children living in dangerous environments to enable them to access health services and to protect them from gender and social violence?***

***Mr. X3:*** we work with the chairpersons, because they are the right people to provide us with social information, we do not even use teachers to provide us with child information because they spend more time at school than at home.

***Questioner: are the available opportunities consistent with the needs of children living in vulnerable environments in enabling them to access health services and protect them from gender and social violence?***

***Mr. X3:*** the available opportunities are small, for example, you find that a family has many children and the insurances come out to sponsor two children, so others are lacking, there are also insurances like CHF that would be better because the process is not very long like NHIF.

***Mr. X1:*** all the opportunities meet the needs of the needy and help them in their families progress especially economic progress, there are good progresses concerning the children who had given up with school, and now are back and are progressing well academically.
