# Supplementary material for: Challenges in accessing health care and socio-protection services among children living and working in streets in northwestern Tanzania: A qualitative study
Source: PLOS Glob Public Health. 2023 May 17;3(5):e0001916. doi: 10.1371/journal.pgph.0001916 (PMC10191300; doi:10.1371/journal.pgph.0001916)
Supplement: S1 Data — (ZIP) [file pgph.0001916.s001.zip › Data/HOSPITAL 2.docx]

Interview guide for X2 hospital

**Muulizaji:** je ni njia hasa mnazitumia katika kuhakikisha watoto wanaoishi na kufanya kazi mtaani wanapata huduma za afya na kuwalindwa dhidi ya ukatili wa kijinsia na kijamii?

**Dr. X1:** sisi kama kituo cha afya jukumu letu ni kwapokea hao watoto na kutoa huduma za kitabibu either bure na kama anamsaada pia tunamuhudumia.

**Muulizaji:** je mnatambuaje kuwa huyo mtoto anamsaada kama amekuja mwenyewe?,na mnatoahuduma kama amefika mwenyewe?

**Dr. X1:** tutaangalia kama amefika mwenyewe tutamuhudumia ila kama amefika na mtu aweza kuwa jirani, ndugu au mtu yeyote kuna gharama lazima atalipia.

**Muulizaji:** kwa sasa ni mashirika mangapi yaliyopo katika wilaya ya Nyamagana na mnashirikiana nayo katika kuwasaidia watoto wanaoishi na kufanya kazi mtaani?

**Dr. X1:** kwa sasa ni ngumu kujua takwimu ya mashirika tuliyoshirikiana nayo maana wao wanatuita mara chache sana na sio mara kwa mara, sisi tupo tayari kwenda kwenye mashirika lkn mara nyingi mashrika yanatuita kufanya huduma ya screening na kuhudumia kama kuna mtoto anaumwa endapo kama anauhitaji wa kulazwa tunampeleka hospital. Hivyo tunavyoshirikiana na mashirika.

**Muulizaji:** ni nini hasa majukumu yenu hususani katika mashirika yanayojihusisha kusaidia watoto wanaoishi na kufanya kazi mitaani?

**Dr. X1:** kutoa matibabu pale ninapohitajika, kutoa elimu kuhusu kujua, kuzuia ukatili wa kijinsia katika jamii.

**Muulizaji:** je mashirika haya yanayojihusisha na watoto wanaoishi na kufanya kazi mitaani yanatakiwa kufanya nini hasa katika kuwasaidia kupata huduma ya afya?

**Dr. X1:** kwanza wawe waminifu na watekeleze majukumu yale yaliowekwa katika mashirika na kuandaa budget ambazo zitawasaidia watoto kupata huduma ya afya, na forming parternship na hospitals ili kuwepo na urahisi wa watoto kupata huduma za afya.

**Muulizaji:** je ni misaada ipi mnaitoa katika mashirika haya ya kijamii kuwasaidia watoto wanaoishi na kufanya kazi mitaani kupata huduma za afya na kuwalinda dhidi ya ukatili wa kijinsia na kijamii?

**Dr. X1:** tunatoa exemption ya kutoa huduma ya afya kwa watoto. Tunatoa materials soaps, mafuta ya kupaka mwilini, dawa pale zinapohitajika.

**Muulizaji:** Je ni vikwazo vipi hasa ambavyo mnakutana navyo kutoka mashirika yanoyotoa msaada kwa watoto wanaoishi na kufanya kazi mitaani?

**Dr. X1:** mashirika mengi tunapokuwa tumeweka appointment nao wanakuwa hawapo on time, pesa ninayolipwa haiendani na gharama za vifaa matibabu pale tunapohitajika kuwasaidia hao watoto.

**Muulizaji:** je nichangamoto zipi hasa ambazo mnakutana nazo katika kuhakikisha watoto wanaoishi na kufanya kazi mitaani wanapata huduma za afya na kulindwa dhidi ya ukatili wa kijinsia na kijamii?

**Dr. X1:** hapana.

**Muulizaji:**  je ni vikwazo vipi mnakutana navyo kwenye jamii inayowazinguka hao watoto wanaoishi na kufanya kazi mitaani katika kuhakikisha wanapata huduma za afya?

**Dr. X1:** watu hawana uelewa na ushirikiano na sisi kama taasisi za afya kwakutambua kuwa wanaweza kupata msaada kutoka kwetu, inaweza kuwa wanachangamoto kutufikia ili wapate huduma.

**Muulizaji:** je ni vikwazo vipi mnakutana navyo kwenye mashirika haya yanayojihusisha na kuwasaidia watoto wanaoishi na kufanya kazi mitaani katika kuhakikisha wanapata huduma za afya?

**Dr. X1:** kikwazo ni kimoja tu time management inapelekea kughaili ratiba zako.

**Muulizaji:** je ni fursa zipi zilizopo katika kuwasaidia watoto wanaoishi na kufanya kazi mitaani katika kupata huduma za afya na kuwalinda dhidi ya ukatili wa kijinsia na kijamii?

**Dr. X1:** Taasisi yetu inapopata fursa ya kuitwa na mashirika malipo yale yanaongeza mapato kwenye kituo chetu.

**MWISHO**
