# Supplementary material for: Challenges in accessing health care and socio-protection services among children living and working in streets in northwestern Tanzania: A qualitative study
Source: PLOS Glob Public Health. 2023 May 17;3(5):e0001916. doi: 10.1371/journal.pgph.0001916 (PMC10191300; doi:10.1371/journal.pgph.0001916)
Supplement: S1 Data — (ZIP) [file pgph.0001916.s001.zip › Data/HW HOSPITAL ENGLISH.docx]

**INTERVIEW GUIDE FOR HOSPITAL**

***Interview with HW from A Hospital popularly XXXX***

***Questioner: what exactly are your responsibilities as a social organization here in the city of Mwanza?(how are you involved when it comes to street children)***

**HW:** Our main responsibilities are to donate open shoes and bites like biscuits whenever they come here, and there are children who are being educated by our institution, which was established by the late Dr. Chacha and are still going to school and others have completed their studies. We also provide all the health services to the street children for free, but protecting them from cruel acts is difficult because we spend most of the time in the hospital and after that we return home to continue our duties as mothers.

***Questioner: what exactly are you doing to help children who live and work on the streets to get health care?***

***HW:*** the children come on their own since they know they can have access to the health services, all they have to do is express themselves and receive treatment.

***Questioner: What methods do you use to ensure that children who live and work in the street are protected against sexual and social violence?***

**HW:** we don't have any method other than providing medical care and striving for their health to be safe.

***Questioner: how many children living and working on the streets do you help in enabling them to access health services? And how many of them are women?***

**HW:** we do not have the exact statistics showing the number of children who received treatment because there are many children and there is no charge when they come here to be treated.

***Questioner: What aid do you provide as a social organization in helping children who live and work on the streets to get health services and protect them from sexual and social violence?***

**HW:** due to the current situation, the center can't manage to help the children financially, but we always give once in a while, we give open shoes and bites like biscuits.

***Questioner: are there any organizations that you cooperate with, in helping and getting health care for children who live and work on the streets?***

**HW:** no, and we are ready to cooperate with organizations in enabling these children to get food.

***Questioner: what are the obstacles you encounter as an organization when you provide help to children living and working on the streets?***

**HW:** we have received a complaint that when these children are admitted to the sick ward, they tend to steal from other patients' belongings, so we decided to separate them from other patients but once done with the treatment they leave, another challenge is improper dose usage, it leads to the child's body weakness, the children report that the medicines are taken by the older boys.

***Questioner: What are the opportunities available to help children living and working on the streets and in accessing health services and protecting them from sexual violence?***

**HW:** No we haven't had any opportunity yet.
