# Supplementary material for: Challenges in accessing health care and socio-protection services among children living and working in streets in northwestern Tanzania: A qualitative study
Source: PLOS Glob Public Health. 2023 May 17;3(5):e0001916. doi: 10.1371/journal.pgph.0001916 (PMC10191300; doi:10.1371/journal.pgph.0001916)
Supplement: S1 Data — (ZIP) [file pgph.0001916.s001.zip › Data/interview guide english version.docx]

**Interview Guide for CSO – Social Welfare Departments**

**A. Responsibilities in Providing Street Children with Health Services and Protecting Them against Gender-Based Violence**

1. What methods do you use to ensure that children who live and work on the streets have access to health services and are protected against sexual and social violence?
2. Are there social organizations that work to help children who live and work on the streets in enabling them have access to health services?

**Questionnaire:** What are those organizations?

**Questionnaire:** What about protecting them against sexual and social violence?

**Questionnaire:** What are those organizations?

1. Currently, how many organizations in your district are involved in helping children living and working in the streets?

**Questionnaire:** how many of these organizations are involved in helping children living and working in the streets in accessing health services?

**Questionnaire:** how many of these organizations are involved in helping children living and working on the streets against sexual violence?

1. What are your responsibilities, especially in organizations involved in helping children living and working on the streets?

**Questionnaire:** What are your particular responsibilities in ensuring that children who live and work on the streets get health services and are protected against sexual and social violence?

1. How can these organizations involved with children living and working on the streets supposed to help these children?

**Questionnaire:** What exactly should they do to help Children who live and work on the streets get health care?

**Questionnaire:** What exactly should they do to protect children living and working on the streets from sexual violence and violent acts?

1. What aid do you provide in these social organizations to help children who live and work on the streets to get health services and protect them from sexual and social violence?

**B. Obstacles and Challenges in Providing Street Children with Health Services and protecting them against sexual and social Violence**

1. What are the specific obstacles that you encounter from organizations that provide support to children living and working on the streets?
2. What challenges do you find in your activities from social organizations that deal with children living and working on the streets?
3. What are the particular challenges that you encounter in ensuring that children who live and work on the streets have access to health services and are protected against sexual and social violence?
4. What obstacles do you encounter in these organizations involved in helping children who live and work on the streets in ensuring they get health services?
5. What obstacles do you encounter in these organizations involved in helping children living and working on the streets to ensure they are protected from sexual and social violence?

**C. Opportunities and Chances in Providing Street Children with Health Services and protecting them against sexual and Social Violence**

1. What are the opportunities available to help children living and working on the streets in accessing health services and protecting them from sexual and social violence?

**Questionnaire:** What methods do you use to find or get those opportunities?

**Questionnaire:** What methods do you use to ensure that the opportunities that arise reach the children who live and work on the streets to enable them to access health services and to protect them from sexual and social violence?

1. Are the opportunities available compatible with the needs of children living and working on the streets in enabling them to access Health services and Protect them against sexual and Social Violence?
2. How do you work with the health Departments, together with the health Centers in ensuring that Children who live and work on the streets get health services and are protected against sexual and social violence?
3. How do you work with the surrounding community to ensure that children who live and work on the streets have access to health services and are protected against sexual and social violence?

**Interview Guide for CSO**

**Guide to interviews with Social Organizations on Access to Health Services and protecting children living and working in the streets Against Sexual Violence in the Community.**

1. What exactly are your responsibilities as a social organization here in Mwanza?

**Questionnaire:** how do you get involved with children who live and work in the street?

2. What are your particular responsibilities in helping children who live and work on the streets?

**Questionnaire:** What are your particular responsibilities in ensuring that children who live and work on the streets get health services and are protected against sexual and social violence?

3. What exactly are you doing to help these children?

**Questionnaire:** What exactly are you doing to help children who live and work on the streets to get health care?

**Questionnaire:** What exactly are you doing to protect children living and working on the streets from sexual violence and violent acts?

4. What methods do you use to ensure that children who live and work on the streets have access to health services?

5. What are the methods you use to ensure that children living and working on the streets are protected from sexual and social violence?

6. How many children living and working on the streets do you help in enabling them to get health services?

**Questionnaire:** What about protecting them against sexual and social violence?

**Questionnaire:** How many of them are women?

7. What aid do you provide as a social organization in helping children who live and work on the streets to get health services and protect them from sexual and social violence?

**D. Obstacles and Challenges in Providing Street Children with Health Services and protecting them against social and sexual Violence**

1. What are the specific obstacles that you encounter as an organization when you provide support to children who live and work on the streets?

2. What are the specific challenges you encounter in your activities as a social organization dealing with children living and working on the streets?

3. What are the particular challenges that you encounter in ensuring that children who live and work on the streets have access to health services and are protected against sexual and social violence?

4. What are the obstacles you encounter as an organization involved in helping children living and working on the streets to ensure they get health services?

5. What are the obstacles you encounter as an organization involved in helping children living and working on the streets to ensure they are protected against sexual and social violence?

6. How do you work with the Departments of Health, together with the health Centers in ensuring that Children who live and work on the streets have access to health services and are protected against sexual and social violence?

7. How do you work with the surrounding community in ensuring children living and working on the streets get health services and are protected against sexual and social violence?

**E. Opportunities in Providing Street Children with Health Services and protecting them against sexual and Social Violence**

1. What are the opportunities available to help children living and working on the streets in accessing health services and protecting them from sexual and social violence?

**Questionnaire:** What methods do you use to find or get those opportunities?

**Questionnaire:** What methods do you use to ensure that emerging opportunities reach Children living and working on the streets to enable them to access Health services and to protect them from sexual and social violence?

2. Are the opportunities available compatible with the needs of children living and working on the streets in enabling them to access Health services and Protect them against sexual and Social Violence?

**Interview Guide for CLWS**

**A. Questions for Children who live and work in the streets**

| **S/N** | | **Personal details** |  |
| --- | --- | --- | --- |
| 1 | | Place of residence |  |
| 2 | | Age |  |
| 3 | | Gender | 1. Male 2. Female |
| 4 | | Tribe |  |
| 5 | | Religion | 1. Christian 2. Muslim 3. Others |
| 6 | | Education | 1. Never studied 2. Never finished 3. Primary level 4. Secondary level |
| 7 | | The kind of job you do in the streets |  |
| 8 | | Has parents? | 1. Yes 2. No 3. Has one (state the gender) |
| 9 | | Reasons for living and working in the streets. |  |
| 10 | | When did you start living in the streets? |  |
| 11 | | Have you ever fallen sick even once? | 1. Yes 2. No |
| 12 | | Have you ever been treated in a health care facility such as a hospital, clinic, etc. | 1. Yes 2. No   If YES, where exactly?_____________________ |
| 13 | | When you went to be treated, who covered or paid for your medical expenses? |  |
| 14 | | Where do you usually get medical help when you get sick? | 1. Yes 2. No   If YES, How did you know or how did you manage to get that help?  _________________________________________ |
| 15 | | Do you know anything concerning sexual violence? | 1. Yes 2. No   If YES please state briefly how you understand sexual violence:  _____________________________________________  _____________________________________________ |
| 16 | | Have you ever experienced any of the following? | \| **Have you ever experienced any of the following?** \| Yes \| Hapana \| \| --- \| --- \| --- \| \| 1. Have you ever been raped/sodomised? \|  \|  \| \| 1. Have you ever been injured? \|  \|  \| \| 1. Have you ever been touched in an inappropriate way? \|  \|  \| \| 1. Have you ever been punished without any reason? \|  \|  \| \|  \|  \|  \| \|  \|  \|  \| |
| 17 | | Do you remember who did that cruelty to you? |  |
| 16 | | Where did you get your knowledge or understanding concerning sexual violence? |  |
| 16 | | Have you ever been treated with any cruelty? | 1. Yes 2. No   If YES, what kind of cruelty was that?_______________________  Where did you report the cruelty after you experienced it?  __________________________________________________ |
| 17 | | Have you ever witnessed or seen another child being treated with any cruelty? | 1. Yes 2. No   If YES, what kind of cruelty was that?_______________________  After witnessing that, what was your reaction/solution?  __________________________________________________ |
| 18 | | Did you get any help when you were subjected to sexual violence? | 1. Yes 2. No?_______________________   If YES, what kind of help was that? |
| 19 | | When you experienced the sexual violence who helped you? |  |
| 20 | | Where do you get help when you experience or are subjected to sexual violence? |  |
| 21 | What challenges do you face when you go to the hospital or health care centers? | |  |
| 22 | Are there any problems or challenges you get from the patients at the hospital? | |  |
| 23 | Are there any problems or challenges you get from doctors or nurses at the hospital? | |  |
| 24 | What exactly causes children to steal things from patients they find in the hospital? | |  |

**FINISH**
