# Supplementary material for: Challenges in accessing health care and socio-protection services among children living and working in streets in northwestern Tanzania: A qualitative study
Source: PLOS Glob Public Health. 2023 May 17;3(5):e0001916. doi: 10.1371/journal.pgph.0001916 (PMC10191300; doi:10.1371/journal.pgph.0001916)
Supplement: S1 Data — (ZIP) [file pgph.0001916.s001.zip › Data/Interview Guide for CSO_1.docx]

*Interview Guide for CSO – Social Welfare Departments*

1. **Majukumu katika Kuwapatia Watoto Waishio Mtaani Huduma za Afya na Kuwalinda Dhidi ya Ukatili wa Kijinsia wa Kijamii**

**A. Responsibilities in Providing Street Children with Health Services and Protecting Them against Gender-Based Violence**

1. Je, ni njia zipi hasa mnazitumia katika kuhakikisha watoto wanaoishi na kufanya kazi mitaani wanapata huduma za Afya na kulindwa dhidi ya ukatili wa kijinsia na kijamii?

*1. What methods do you use to ensure that children who live and work on the streets have access to health services and are protected against sexual and social violence?*

1. Je kuna mashirika ya kijamii ambayo yanafanya kazi ya kusaidia watoto wanaoishi na kufanya kazi mitaani katika kuwawezesha kupata huduma za afya?

*2. Are there social organizations that work to help children who live and work on the streets in enabling them have access to health services?*

***Dodosa:*** Je ni mashirika yapi hayo?

*Question: What are those organizations?*

***Dodosa:*** Je katika kuwalinda dhidi ya ukatili wa kijisnia na kijamii?

*Question: What about protecting them against sexual and social violence?*

***Dodosa:*** Je ni mashirika yapi hayo?

*Question: What are those organizations?*

1. Kwasasa ni mashirika mangapi yaliyopo wilayani kwako yanajihusisha na kusaidia Watoto wanaoishi na kufanya kazi mitaani.

*3. Currently, how many organizations in your district are involved in helping children living and working on the streets.*

***Dodosa:*** *je, katika mashirika haya, ni mangapi yanajihusisha na kuwasaidia* watoto wanaoishi na kufanya kazi mitaani katika kupata huduma za afya?

*Question: how many of these organizations are involved in helping children living and working on the streets in accessing health services?*

***Dodosa:*** *je, katika mashirika haya, ni mangapi yanajihusisha na kuwasaidia* watoto wanaoishi na kufanya kazi mitaani dhidi ya ukatili wa kijinsia?

*Question: how many of these organizations are involved in helping children living and working on the streets against sexual violence?*

1. Ni nini hasa majukumu yenu hususani katika mashirika yanayojihusisha kusaidia watoto wanaoishi na kufanya kazi mitaani.

*4. What are your responsibilities, especially in organizations involved in helping children living and working on the streets.*

***Dodosa:*** *Je majukumu yenu hasa ni yapi katika kuhakikisha* watoto wanaoishi na kufanya kazi mitaani wanapata huduma za afya na kulindwa dhidi ya ukatili wa kijinsia na kijamii?

*Question: What are your particular responsibilities in ensuring that children who live and work on the streets get health services and are protected against sexual and social violence?*

1. Je, mashirika haya yanayojihusisha na Watoto wanaoishi na kufanya kazi mitaani yanatakiwa kufanya nini hasa katika kuwasaidia watoto hawa?
2. *How can these organizations involved with children living and working on the streets supposed to help these children?*

***Dodosa:*** Ni nini haswa yanapaswa kufanya katika kuwasaidia Watoto wanaoishi na kufanya kazi mitaani kupata huduma ya afya?

*Question: What exactly should they do to help Children who live and work on the streets get health care?*

***Dodosa:*** Ni nini haswa yanapaswa kufanya katika kuwalinda watoto wanaoishi na kufanya kazi mitaani dhidi ya unyanyasaji wa kijinsia na vitendo vya kikatili?

*Question: What exactly should they do to protect children living and working on the streets from sexual violence and violent acts?*

1. Je, ni misaada ipi mnaitoa katika mashirika haya ya kijamii kuwasaidia watoto wanaoishi na kufanya kazi mitaani kupata huduma za Afya na kuwalinda dhidi ya ukatili wa kijinsia na Kijamii?

*6. What aid do you provide in these social organizations to help children who live and work on the streets to get health services and protect them from sexual and social violence?*

1. **Vikwazo na Changamoto katika Kuwapatia Watoto Waishio Mtaani Huduma za Afya na Kuwalinda Dhidi ya Ukatili wa Kijinsia wa Kijamii**

***B. Obstacles and Challenges in Providing Street Children with Health Services and Protecting Them against sexual and social Violence***

1. Je, ni vikwazo vipi hasa ambavyo mnakutana navyo kutoka katika mashirika yanayotoa msaada kwa Watoto wanaoishi na kufanya kazi mitaani?

*1. What are the specific obstacles that you encounter from organizations that provide support to children living and working on the streets?*

1. Je. nichangamoto zipi hasa mnazozipata katika shughuli zenu kutoka katika mashirika ya kijamii yanayohusika na Watoto wanaoishi na kufanya kazi mitaani.

*2. What challenges do you find in your activities from social organizations that deal with children living and working on the streets.*

1. Je, ni **Changamoto** zipi hasa ambazo mnakutana nazo katika kuhakikisha watoto wanaoishi na kufanya kazi mitaani wanapata huduma za Afya na Kulindwa dhidi ya Ukatili wa Kijinsia na Kijamii?

3. What are the particular challenges that you encounter in ensuring that children who live and work on the streets have access to health services and are protected against sexual and social violence?

1. ***J****e, ni vikwazo vipi mnakutana navyo kwenye mashirika haya yanajihusisha na kuwasaidia* watoto wanaoishi na kufanya kazi mitaani katika kuhakiksha wanapata huduma za afya?

*4. What obstacles do you encounter in these organizations involved in helping children who live and work on the streets in ensuring they get health services?*

1. Je, ni vikwazo vipi mnakutana navyo kwenye mashirika haya yanajihusisha na kuwasaidia watoto wanaoishi na kufanya kazi mitaani katika kuhakikisha wanalindwa dhidi ya ukatili wa kijinsia na kijamii?

*5. What obstacles do you encounter in these organizations involved in helping children living and working on the streets to ensure they are protected from sexual and social violence?*

1. **Fursa na Nafasi katika Kuwapatia Watoto Waishio Mtaani Huduma za Afya na Kuwalinda Dhidi ya Ukatili wa Kijinsia wa Kijamii**

***C. Opportunities and Chances in Providing Street Children with Health Services and protecting them against sexual and Social Violence***

1. Je, ni fursa zipi zilizopo katika kuwasaidia watoto wanaoishi na kufanya kazi mitaani katika kupata huduma za Afya na kuwalinda dhidi ya ukatili wa kijinsia na Kijamii?

*1. What are the opportunities available to help children living and working on the streets in accessing health services and protecting them from sexual and social violence?*

***Dodosa:*** Je mnatumia mbinu gani kutafuta ama kupata fursa hizo?

*Question: What methods do you use to find or get those opportunities?*

***Dodosa:*** Je mnatumia mbinu zipi kuhakikisha fursa zinazojitokeza zinawafikia watoto wanaoishi na kufanya kazi mitaani ili kuwawezesha kupata huduma za Afya na katika kuwalinda dhidi ya ukatili wa kijinsia na Kijamii?

*Questionnaire: What methods do you use to ensure that the opportunities that arise reach the children who live and work on the streets to enable them to access health services and to protect them from sexual and social violence?*

1. Je fursa zinazopatikana zinaendana na mahitaji ya watoto wanaoishi na kufanya kazi mitaani katika kuwawezesha kupata huduma za Afya na Kuwalinda dhidi ya Ukatili wa kijinsia na Kijamii?

*2. Are the opportunities available compatible with the needs of children living and working on the streets in enabling them to access Health services and Protect them against sexual and Social Violence?*

1. Je, ni kwanamna gani mnafanya kazi na Idara za afya, Pamoja na Vituo vya kutolea huduma za afya katika kuhakikisha Watoto wanaoishi na kufanya kazi mitaani wanapata huduma za afya na kulindwa dhidi ya ukatili wa kijinsia na kijamii?

*3. How do you work with the health Departments, together with the health Centers in ensuring that Children who live and work on the streets get health services and are protected against sexual and social violence?*

1. Je ni kwanamna gani mnafanya kazi na jamii inayowazunguka katika kuhakikisha Watoto wanaoishi na kufanya kazi mitaani wanapata huduma za Afya na kulindwa dhidi ya ukatili wa kijinsia na Kijamii?

*4. How do you work with the surrounding community to ensure that children who live and work on the streets have access to health services and are protected against sexual and social violence?*

Interview Guide for CSO

***Mwongozo wa mahojiano na Mashirika ya Kijamii kuhusu Upatikanaji* Huduma za Afya na Kuwalinda Dhidi ya Ukatili wa Kijinsia wa Kijamii kwa Watoto Waishio na kufanya kazi Mitaani**

***Guide to interviews with Social Organizations on Access to Health Services and protecting children living and working in the streets Against Sexual Violence in the Community.***

1. Je ni nini hasa majukumu yenu kama shirika la kijamii hapa Jijini Mwanza?

*1. What exactly are your responsibilities as a social organization here in Mwanza?*

***Dodosa:*** je ni kwanamna gani mnajihusisha na watoto waishio na kufanya kazi mtaani?

*Questionnaire: how do you get involved with children who live and work in the street?*

1. Ni nini majukumu yenu hasa hususani katika kujishughulisha na kusaidia watoto wanaoishi na kufanya kazi mitaani?

*2. What are your particular responsibilities in helping children who live and work on the streets?*

***Dodosa:*** *Je majukumu yenu hasa ni yapi katika kuhakikisha* watoto wanaoishi na kufanya kazi mitaani wanapata huduma za afya na kulindwa dhidi ya ukatili wa kijinsia na kijamii?

*Question: What are your particular responsibilities in ensuring that children who live and work on the streets get health services and are protected against sexual and social violence?*

1. Je, mnafanya nini hasa katika kuwasaidia watoto hawa?

*3. What exactly are you doing to help these children?*

***Dodosa:*** Ni nini haswa mnafanya katika kuwasaidia Watoto wanaoishi na kufanya kazi mitaani kupata huduma ya afya?

*Questionnaire: What exactly are you doing to help children who live and work on the streets to get health care?*

***Dodosa:*** Ni nini haswa mnafanya katika kuwalinda watoto wanaoishi na kufanya kazi mitaani dhidi ya unyanyasaji wa kijinsia na vitendo vya kikatili?

*Questionnaire: What exactly are you doing to protect children living and working on the streets from sexual violence and violent acts?*

1. Je, mnatumia mbinu gani kuhakikisha watoto wanaoishi na kufanya kazi mitaani wanapata huduma za Afya?

4. *What methods do you use to ensure that children who live and work on the streets have access to health services?*

1. Je, ni njia zipi hasa mnazitumia katika kuhakikisha watoto wanaoishi na kufanya kazi mitaani wanalindwa dhidi ya ukatili wa kijinsia na kijamii?

*5. What are the methods you use to ensure that children living and working on the streets are protected from sexual and social violence?*

1. Je ni Watoto wangapi wanaoishi na kufanya kazi mitaani mnawasaidia katika kuwawezesha kupata huduma za afya?

*6. How many children living and working on the streets do you help in enabling them to get health services?*

***Dodosa:*** Je katika kuwalinda dhidi ya ukatili wa kijinsia na kijamii?

Questionnaire: What about protecting them against sexual and social violence?

***Dodosa:*** Je kati yao wa wakike ni wangapi?

Question: How many of them are women?

1. Je, ni misaada ipi mnaitoa kama shirika la kijamii katika kuwasaidia watoto wanaoishi na kufanya kazi mitaani kupata huduma za Afya na kuwalinda dhidi ya ukatili wa kijinsia na Kijamii?

7. What aid do you provide as a social organization in helping children who live and work on the streets to get health services and protect them from sexual and social violence?

1. **Vikwazo na Changamoto katika Kuwapatia Watoto Waishio Mtaani Huduma za Afya na Kuwalinda Dhidi ya Ukatili wa Kijinsia wa Kijamii**

***D. Obstacles and Challenges in Providing Street Children with Health Services and protecting them against social and sexual Violence***

1. Je, ni vikwazo vipi hasa ambavyo mnakutana navyo kama shirika mnapokuwa mkitoa msaada kwa Watoto wanaoishi na kufanya kazi mitaani?

1. What are the specific obstacles that you encounter as an organization when you provide support to children who live and work on the streets?

1. Je, nichangamoto zipi hasa mnazozipata katika shughuli zenu kama shirika la kijamii mnaohusika na Watoto wanaoishi na kufanya kazi mitaani?

*2. What are the specific challenges you encounter in your activities as a social organization dealing with children living and working on the streets?*

1. Je, ni **Changamoto** zipi hasa ambazo mnakutana nazo katika kuhakikisha watoto wanaoishi na kufanya kazi mitaani wanapata huduma za Afya na Kulindwa dhidi ya Ukatili wa Kijinsia na Kijamii?

3. What are the particular challenges that you encounter in ensuring that children who live and work on the streets have access to health services and are protected against sexual and social violence?

1. Je, ni vikwazo vipi mnakutana navyo kama shirika manaojihusisha na kuwasaidia watoto wanaoishi na kufanya kazi mitaani katika kuhakikisha wanapata huduma za afya?

4. What are the obstacles you encounter as an organization involved in helping children living and working on the streets to ensure they get health services?

1. Je, ni vikwazo vipi mnakutana navyo kama shirika manaojihusisha na kuwasaidia watoto wanaoishi na kufanya kazi mitaani katika kuhakikisha wanalindwa dhidi ya ukatili wa kijinsia na kijamii?

*5. What are the obstacles you encounter as an organization involved in helping children living and working on the streets to ensure they are protected against sexual and social violence?*

1. Je, ni kwanamna gani mnafanya kazi na Idara za afya, Pamoja na Vituo vya kutolea huduma za afya katika kuhakikisha Watoto wanaoishi na kufanya kazi mitaani wanapata huduma za afya na kulindwa dhidi ya ukatili wa kijinsia na kijamii?

*6. How do you work with the Departments of Health, together with the health Centers in ensuring that Children who live and work on the streets have access to health services and are protected against sexual and social violence?*

1. Je ni kwanamna gani mnafanya kazi na jamii inayowazunguka katika kuhakikisha Watoto wanaoishi na kufanya kazi mitaani wanapata huduma za Afya na kulindwa dhidi ya ukatili wa kijinsia na Kijamii?

*7. How do you work with the surrounding community in ensuring children living and working on the streets get health services and are protected against sexual and social violence?*

1. **Fursa na Nafasi katika Kuwapatia Watoto Waishio Mtaani Huduma za Afya na Kuwalinda Dhidi ya Ukatili wa Kijinsia wa Kijamii**

**E. Opportunities in Providing Street Children with Health Services and Protecting Them Against sexual and Social Violence**

1. Je, ni fursa zipi zilizopo katika kuwasaidia watoto wanaoishi na kufanya kazi mitaani katika kupata huduma za Afya na kuwalinda dhidi ya ukatili wa kijinsia na Kijamii?

2. What are the opportunities available to help children living and working on the streets in accessing health services and protecting them from sexual and social violence?

***Dodosa:*** Je mnatumia mbinu gani kutafuta ama kupata fursa hizo?

Questionnaire: What methods do you use to find or get those opportunities?

***Dodosa:*** Je mnatumia mbinu zipi kuhakikisha fursa zinazojitokeza zinawafikia

watoto wanaoishi na kufanya kazi mitaani ili kuwawezesha kupata huduma za Afya na katika kuwalinda dhidi ya ukatili wa kijinsia na Kijamii?

Questionnaire: What methods do you use to ensure that emerging opportunities reach Children living and working on the streets to enable them to access Health services and to protect them from sexual and social violence?

1. Je fursa zinazopatikana zinaendana na mahitaji ya watoto wanaoishi na kufanya kazi mitaani katika kuwawezesha kupata huduma za Afya na Kuwalinda dhidi ya Ukatili wa kijinsia na Kijamii?

6. Are the opportunities available compatible with the needs of children living and working on the streets in enabling them to access Health services and Protect them against sexual and Social Violence?

Interview Guide for CLWS

1. **Maswali kwa Watoto watoto wanaoishi na kufanya kazi mitaani**

| S/N | | Taarifa Binafsi |  |
| --- | --- | --- | --- |
| 1 | | Mahali alipokuwa anaishi |  |
| 2 | | Umri |  |
| 3 | | Jinsia | 1. Mvulana 2. Msichana |
| 4 | | Kabila |  |
| 5 | | Dini | 1. Mkiristo 2. Muislam 3. Nyingine |
| 6 | | Elimu | 1. Sikusoma 2. Sikumaliza 3. Shule msingi 4. Sekondari |
| 7 | | Kazi afanyayo mtaani |  |
| 8 | | Ana wazazi | 1. Ndiyo 2. Hapana 3. Anaye mmoja (Ainisha Jinsia yake) |
| 9 | | Sababu ya kuishi na kufanya kazi mtaani |  |
| 10 | | Ulianza lini kuishi mtaani |  |
| 11 | | Ulishawahi kuugua hata mara moja | 1. Ndiyo 2. Hapana |
| 12 | | Je, Ulishawahi kutibiwa katika kituo cha kutolea huduma za afya kama hospitali, zahanati n.k | 1. Ndiyo 2. Hapana   Kama, NDIO, Uliwahi kutibiwa wapi_____________________ |
| 13 | | Je, Ulipoenda kutibiwa nani aligharamia ama kukulipia gharama za matibabu? |  |
| 14 | | Je, huwa unapata wapi msaada wa kimatibabu mara kwa mara unapougua? | 1. Ndiyo 2. Hapana   Kama, NDIO, je, ulifahamu vipi ama uliwezaje kuwa unapata huo msaada?_________________________________________ |
| 15 | | Je, unafahamu chochote kuhusu ukatili wa Kijinsia? | 1. Ndiyo 2. Hapana   Kama, NDIO, elezea kwa ufupi kuhusu ukatili wa kijinsia _____________________________________________  Toa Mifano: _____________________________________________ |
| 16 | | Je, ulishawahi kutendewa jambo lolote kati ya haya yafuatayo | \| **Je, ulishawahi kutendewa jambo lolote kati ya haya yafuatayo** \| Ndio \| Hapana \| \| --- \| --- \| --- \| \| 1. Ulishawahi kubakwa/kulawitiwa \|  \|  \| \| 1. Ulishawahi kujeruhiwa \|  \|  \| \| 1. Kupapaswa \|  \|  \| \| 1. Kuadhibiwa bila kosa \|  \|  \| \| 1. Ulishawahi kubakwa/kulawitiwa \|  \|  \| \| 1. Ulishawahi kujeruhiwa \|  \|  \| |
| 17 | | Je, unakumbuka ninani alikutendea ukatili huo? |  |
| 16 | | Je ulipata wapi elimu ama uelewa kuhusu ukatili wa kijinsia? |  |
| 16 | | Je, Ulishawahi kutendewa ukatili wowote? | 1. Ndiyo 2. Hapana   Kama, NDIO, je, ulitendewa Ukatili upi?_______________________  Je baada ya kutendewa ukatili huo ulienda kulitoa taarifa wapi?  __________________________________________________ |
| 17 | | Je, Ulishawahi kushuhudia ma kuona mtoto mwingine akitendewa ukatili wowote? | 1. Ndiyo 2. Hapana   Kama, NDIO, je, alitendewa Ukatili upi?_______________________  Je baada ya kutendewa ukatili huo ulifanya nini?  __________________________________________________ |
| 18 | | Je ulipotendewa ukatili wa kijinsia ulipata msaada wowote? | 1. Ndiyo 2. Hapana   Kama, NDIO, je, ulipata msaada upi?_______________________ |
| 19 | | Je, Ulipotendewa ukatili huo nani alikusaidia? |  |
| 20 | | Je, huwa unapata wapi msaada mara unapokutana ama kutendewa ukatili wa kijinsia? |  |
| 21 | Je, Mnapata changamoto gani mnapokwenda hospitali (vituo vya kutolea huduma za Afya)? | |  |
| 22 | Je, kuna matatizo yoyote ama changamoto mnayoipata kutoka kwa wagonjwa wa hapo hospitali? | |  |
| 23 | Je, kuna matatizo yoyote ama changamoto mnayoipata kutoka kwa madaktari ama wauguzi-manurse hapo hospitali? | |  |
| 24 | Ni nini hasa husababisha kwa Watoto kudokoa vitu vya wagonjwa wanaowakuta Hospitali? | |  |

**MWISHO**
