# Supplementary material for: Challenges in accessing health care and socio-protection services among children living and working in streets in northwestern Tanzania: A qualitative study
Source: PLOS Glob Public Health. 2023 May 17;3(5):e0001916. doi: 10.1371/journal.pgph.0001916 (PMC10191300; doi:10.1371/journal.pgph.0001916)
Supplement: S1 Data — (ZIP) [file pgph.0001916.s001.zip › Data/railway.docx]

**CSO Y4**

**Response 1:** Railway children is a non-government organization that deals directly with children living in harsh environments especially children living around the streets. We have our offices here in Mwanza but the headquarters are in Dar es Salaam, in addition to that have a project running in Dodoma in partnership with TIZEBET organization, which helps in the implementation of that project. We as Railway Children we allocate money to them to be able to implement that is why we say we are in Dar Es Salaam, Dodoma and Mwanza.

***Questioner: what are your responsibilities as an organization here in Mwanza?***

**Response 1:** As I said earlier, we work with children who live and work on the streets and the activities we do are four major activities, the first activity is street outreach children which we go around various places here in Mwanza where the child is found in Ilemela and Nyamagana districts.

In Ilemela nyamagana district which has 39 districts, there are districts that we are looking at where most of the children are in Nyegezi district. Nyegezi district has a bus stand, so children come in and out when we get there we talk to them. Many children are familiar with Nyegezi and from there they start moving to other areas.

We have worked in Nyegezi and Pamba district for a long time, Natta, Mabatini and Muslim cemeteries where there are many children because it is near the bus stand where children are found in abundance. Nyamagana district links with Kamanga and the city center on the ferries from Sengerema, children leave Bukoba to Kamanga, some prefer to stay there since there is a lake so swimming is free they like it that way. Igogo County, kirumba ward and mwaroni market in Mwanza are places consisting of drunkards, so we always work and go to those places and get information from the children, stake holders and the government itself. There are days we go very early in the morning, there are days during the week we go at night and other days we go during the day and we go to get to know the children, to build a relationship so that they can trust us and work with us. But we also go there to find out their situation. Sometimes you go there and find a sick child, you take him to the hospital. Also, sometimes you find the child is a stranger who has arrived, we need to rescue and take him, then we make efforts to bring him back to their home, sometimes you go there and find them sleeping in the ditches when it rains, it becomes a risky place.

The government also helps us with the children’s progress, in terms where the children stay, sleep, hence we keep on working with them. But also in our street children’s outreach we get support from community members, good Samaritans, food vendors, who have been of great help to the children since we get feedback from the children of the people who helped them, we call them our community champions.

We go to those people, we talk to them, we recognize them, and give them training, we advise them to take the children to the social welfare officer, they mandate the children to find them a place to live, others do not follow the law. They take the children and live with them, which is very dangerous. They should have at least a letter from the Chairman or the police so that if a problem occurs, it will be easier to solve. Sometimes you get a call and you are told that the rangers are beating the children on the streets, so we go there and talk to the police and take them to the welfare officer themselves, they find places for them to stay.

As we are looking for the child’s home, there is always a family that stays with the child for the meantime, once the home is found the child is taken home. So the maximum time for a person to live with the child is six months, after 6 months they go back to the prospective officer telling him that they have stayed with the child and got details so the Street Person and the social Welfare Officer will know the way forward. Then some of us from the streets at night go to the police, the police tend to know where the children are from, so if you go with the police it tends to be more secure since the children found in those areas may threaten the life of our staff member, the good thing the police is always dressed casually so that the children feel free and not frightened.

The second intervention we are doing and it carries Railway children and our hearts and our minds is an organization called family integration or some people like to call it family rule reification. (Reification is to find a child and return them back to their home). We have children here in Mwanza, the majority come from other regions and a few come from Mwanza. Some tend to come due to loss of parents, poverty, adventure and some peer pressure, so the solution cannot be one due to the various reasons each one of them came to the streets.

In the past, we used to think that the child comes from Kigoma, we would take him back to Kigoma, this was time consuming, the children barely gave reliable information, so we decided to do network tracing. We decided that our selected stuffs would go with the child and make follow up concerning their family, through the details provided by the child, for instance knowing the school name, knowing the home surroundings ,we then call the Welfare Officer of the place where the child lived, the Chairman or the Executive until we find the child's family.

Once we find the child's family, we are going to do a household assessment to find out the family health, relationships and economy state and the issues related to sexual violence. We make research in order to know the basic needs of that child, so that when left alone they won’t think of returning to the streets. children living with grandparents, may tell you that they are starving, even if we decide to offer capital to do business to raise their incomes, it would turn out to be a lie since the elderly will not be able to do business, hence we provide child support of that person who will be able to stay with that family.

Families who are self-sufficient, we tend to provide business support as capital and we give them a startup to do business so that they can provide for the needs of their family. We also cooperate with the development officer of the relevant areas so that if possible they can be present in the startup program. We provide CHF insurance which we believe after one year a family can pay for that insurance. So it depends on the needs of the family, and it's not that we shall provide each and every thing, we always talk to stakeholders or the government, they provide equipment, but not to a larger extent, sometimes organizations help us.

Most of the children who come to the streets have two things, either they are enrolled or school dropouts, most are of school age, we are trying to enroll those who are interested and not all, And after the ratification of finding out that the home environment is safe and there is no risk and the parents are ready to receive and raise him, the child will be returned home whether its within or outside Mwanza, in some of the cases he leaves with a social welfare officer to the area that he’s going, the child will be put under the care of the local leadership, the village government and the executive officer, the development officer representative of the school and the neighbors where the child will study will also be met, our remaining job is to make follow-ups of the child and ensure he’s doing great as we continue with other children.

In family work, there is something called parenting, many children come to the streets because of lack of education from their families or training for parents, so when the child reaches the age of adolescent, he starts to show some behavior, the parent sees a solution by striking him/her with a stick, but he does not know that there is a solution. at the age of adolescent at this age the child needs to be helped and that is why we go to the community we do identification gaps finding the different reasons why they are on the streets, so we make arrangements with care takers, families in groups in different areas and provide education called parental child development care for eleven weeks.

We also work with young people in the streets whose priority is not to return home, they like to live their own lives and find themselves involved in risky behaviors, so we meet them in their groups in the places where they are found, we talk to them, we empower them, they calm down, we give them advice so that they can be independent and transform their behavior first. To enable them to gain awareness and calm down. We connect them to vocation training center or other training centers so that they get training and knowledge on how to depend on themselves and not engage on street illegal activities lastly we make prevention on street children previously we were responding on the problem but now we have gone extra miles we make measures. We prepare community dialogues preparing session with the society with our facilities like speaker to the society as organization to educate them concerning on child abuse, child right, how to report cases concerning any abuse cases concerning child's needs how to reduce the number of children in street and avoid cocaine and other drugs, so we call the stakeholders, the Chairman, the Executive and the police so that they can help us take them to the Social Welfare Officer. So we collaborate with stakeholders to prepare a radio program, in short those are the activities we do.

***Questioner: How do you help these children who are going through dangerous environment?***

**Madam Irene:** we rescue the children, we provide them with medical help, if the child has been brutalized, we provide AID and we also protect them in case they are arrested and taken to court, we strive to ensure that the children’s rights are respected. We also connect these children with their families, these are some of the activities we do in the organization.

***Questioner: How many children living on the streets do you help in access of health services?***

**Answer 1:** we don't have a clear number on those that we have helped to access health services, but these young people are under the government, so if he goes to the hospital without money, they cannot provide him with treatment otherwise until he is given documents from Social Welfare. We have agreed that if any child gets sick from the streets, we take him to the AIC Church Hospital, the mission areas, we leave the medicine under the care of a local food vendor for dose management. If a child is raped, we take her to Hope child center or Seokotuore, it's easy since its cheaper,. We provide NHIF cards for about six children who have been on the streets for a long time.

***Questioner: Is there a health care challenge on the part of PF3 for an injured child in the hospital, how do you deal with it?***

**Answer provider 1:** We who know the partners do not find challenges, we take the child to the hospital and they receive easy treatment.

**Questioner:** And among the children living in the harsh environment on the streets are there any female children?

**Answerer 1:** The number of female children is very small compared to male children. It is not very easy for girls to be in the streets. A girl child is marketable in the streets since they can work in local restaurants, they can work as house maids.

***Questioner: What aid do you provide as an organization to help children living on the streets to protect them from health violence?***

**Answerer 1:** We provide education to the children against violence. We often advise them not to sleep in certain places alone, not to be in dark places, not to receive things from strangers. We always escort the children to social workers, since some of them are not confident, hence they can’t easily express themselves. We provide education to adolescent children about sexual reproduction and provide training and HIV/AIDS test for children.

***Questioner: What are the specific obstacles that you encounter as an organization when you provide support to children living and working in the streets?***

**Answer giver 1:** Negative perception, the government believes that we railway children promote children to live in the streets since we provide medical help to the sick children, sick. The NHIF cards we gave them were not working for other treatments such as x-rays, operations and we incurred other costs so that the child could get health support. Cases of sexual violence becomes a challenge in getting the accuser, When a child is raped, we take her to the hospital and gets treatment, but its hard to find the accuser, since they barely remember what happened to them.

Regarding health care, it is very difficult for street children to go to the hospital and they are given help. When you go to the hospital you are told that the X-Ray is not functioning well. Another challenge is that the society does not trust the street children, even if you educate them, they view street children as children different from their own, some people tend to be cruel to them, they are given bad names, sometimes the rangers beat them.

***Questioner: How do you work with health centers as an organization and provide services to children who work in the street and protect them from social violence?***

**Answerer 1:** We have our IDs for street children and the hospital recognizes our IDs, so if a child goes with that ID, he must receive treatment. The hospital that provides the most aid is Seko Toure, there is a social welfare officer, so we get information 24 hours a day.

We have never gone there and failed to get treatment but also the Butimba Hospitals, Nyamagana Makongoro and Buzuruga we have a social welfare officer hence we easily get treatment.But we also recognize the issue of cruelty, we provide trainings at the health facilities so that when there is a case we can have access to PF3 form and with the cases in court, we make sure we fill in the records.

We can clearly see that there was a state prosecutor who was also able to help. But we were also able to hold meetings with inter perception, we gave guidelines on what should be done and he sorts out, for cases of drug addicts, doctors come for training from Bugando and if a problem occurs, we are in parallel with those hospitals.

***Questioner: How do you work with the community surrounded by the street children to protect them against violence?***

**Answerer 1:** Educating the community on how to help children, cooperate with the police and the social welfare officer in hand with the hospital with the services we offer, we also offer health insurance to six children inclusive the father this will help in access of easy treatment.

***Questioner: What opportunities do you offer to help children living and working on the streets by accessing health services and protecting them from sexual violence in society?***

Answerer 1: There are many opportunities provided by the local government and the Community Welfare Officer can provide a permit for free treatment in the hospital for all victims of sexual violence, they can also have access to maternity tests through the support of the government or organizations.

***Questioner: What methods do you use to get those opportunities?***

**Answerer 1:** Being very close to the DMO, we share the title, that there is a day of AIDS, youth day, so we can make contact with the social welfare officer, and then share where the free health services will be provided at a certain area, so they should send the children there. Or the welfare officers who are at the health centers often give us information that there are various opportunities at the hospital that they can squeeze or sometimes the executives give us information that in our ward this week we will have vaccinations for children of this and that age, so you can bring your children they should be vaccinated. For example, during COVID19, children were given sanitizer, masks, soap. Sometimes the executives tell you that this week they will distribute the equipment, so you send the children to get those requirements.

***Questioner: And what method were you using to educate the children since they are scattered?***

**Answerer 1:** We meet them twice a week. You update them concerning each week’s activities. When it comes to oral hygiene, we always encourage them to go for checkups, since we always put tents in play grounds.

Another method we tell local food vendors that when the children come, they should go to the playground there are free health services. Then we tend to have an announcer, who goes around telling children to go to the playground there are free services offered.

***Questioner: Are the opportunities that come out really compatible with their needs?***

**Answer giver 1:** sometimes you find that children have different needs, some have skin diseases, outbreaks of boils, they fight, so we handle them according to their needs.
