# Supplementary material for: Challenges in accessing health care and socio-protection services among children living and working in streets in northwestern Tanzania: A qualitative study
Source: PLOS Glob Public Health. 2023 May 17;3(5):e0001916. doi: 10.1371/journal.pgph.0001916 (PMC10191300; doi:10.1371/journal.pgph.0001916)
Supplement: S1 Data — (ZIP) [file pgph.0001916.s001.zip › Data/STREET CHILDREN.docx]

*SW03*

***Question: what are the methods you use to ensure children living and working in the streets can be protected against sexual and social violence and how do they get health services?***

**Officer G4:** First of all, we don't like to stay during the day, so we can't say that we provide services to street children, but there are those children who come sent by their parents to sell peanuts, bananas, who roam in the streets in the evening and return home, especially during holidays, so even if they get sick, they have families. They tend to work in the streets in the evening and return home, but for health reasons they have their families but there are those who come from another region and decide to sleep. We need to identify them so that we can bring them back home. When we ask them why they have come to the streets, they claim they came to ask for money. Or they came to ask for money to get school supplies, so we want them to return to their homes, we don't want them to stay in the streets because there are challenges in the streets, you can't give them services easily since it’s hard to trace them, they are here today and gone tomorrow.

**Officer G2:** in addition, there are also those children who enter the street who are rescued by Welfare Officers or organizations that deal with street children, so when such children's family details are known, the children are returned to their families but before that, they are sent to health centers for checkup and eventually they are reunited with their families.

***Questioner: Are there social organizations that work to help children who live and work on the streets in enabling them to access health services?***

**Officer G4:** Yes, there are many organizations, one of the biggest organizations is Railway Children, which to a larger percentage work with street children and if they are injured, they take them to the hospital, there is also a health center called CF Hospital, that was serving the children too, Cheka Sana is another organization that gives aid to children and once they are injured they offer help and take them back to their premises.

***Questioner: Currently, how many organizations in your district are involved in helping children who end up working on the streets?***

**Officer G4:** there are three but there are two other small ones which are Farijika, Fanisi, Rafiki for children, Railway children and cheka sana.

***Questioner: How many of these organizations have the power to help these children on the streets?***

**Officer G4:** All agencies

***Questioner: What exactly are your responsibilities, especially in organizations involved in helping street children?***

**Officer G4:** First of all even if the children get the organizations we have to know why they are on the streets and offer advices, we must also make contact with our fellow social welfare officers for example a child says he has come from Ngara district, the responsibility of the social welfare officer in Ngara is to find out the families information, why the child decided to leave, but also to know the health of the child. There is a form we need to fill in order to know how the child's health is, then after we give permission for a child to stay in a shelter or giving them a go ahead to reunite with their families.

**Officer G2:** When we return those children, it is the responsibility of the social Welfare Officer to continue to educate the parents about the whole issue of sending children to work, we educate them on child abuse, sending children to do house works (house maids), sending children to places of work without their wishes, with proper education such cases will not repeat themselves.

***Questioner: Are these organizations involved with children who live and work on the streets, what should they do to help these children?***

**Officer G4:** We often have those social workers, children tend to lie if you talk to them harshly, so organizations should make friends with those children and have a social worker whom they can call a teacher, there are cases you need to talk to the child for about a month, so that they have confidence in you and they can open up, but if you meet the child on the first day, he may not open up. If he’s new to the streets he may get tired and feel guilty hence may go back home. but in cases a child spends a lot of time in the streets, friendship is critically needed, the good part is that the organizations tends to have employees who are in the streets every day, they are close to the children, they prepare and bring them to us in order to get them the other basic needs that were offered to us.

There is also another organization called Upendo Daima. They have their own center where children stay with them. Sometimes you find that they take them and go to stay with them. The children tend to have experienced harsh living environments, The same organizations provides them with school supplies but also talks to them, and gives some money for upkeep.

**Officer G2:** sometimes they help the parents of the children who came here, the children leave their families, and their income becomes difficult, they give them money for capital to increase their income, sometimes the children leave their homes because of the income.

***Interviewer: What aid do you provide from these social organizations to help children living and do street work to access health care and protect them from sexual violence?***

**Officer G4:** in organizations we work together with them, but also sometimes a child can get hurt, organizations don't have money, we appeal, they go to our hospitals and receive treatment. We work side by side with them in case of any issues they consult us since they cannot work without involving us.

**Officer G4:** Even the medical forms come from Community Welfare.

***Questioner: What are the specific obstacles you encounter from organizations that are responsible for providing support to children living and working on the streets?***

**Officer G4:** There are not many obstacles because they help us without them we wouldn't be able to. They help us find the children, there are no major obstacles. We live in peace and harmony. The challenges are with the street children, not for the organizations, the children do not give full cooperation, even the ones you see in the streets they have gone through different organizations, we have taken them to their parents but after few months they head back to the streets. They tend to make jokes and call us names like madam, as if they know you from a place, these are some of the challenges.

**Officer G4:** Another challenge we face is that the children we are involved with do not give full information because he could have stolen or done something bad at their place and now that is bothering us, they tend to give us unreliable information.

**Officer G4:** Another challenge is that we tend to put them under the care of our trusted person, to take care of them as they wait to get back home. They tend to rob our people who have left something of value, either money or bags of clothes, so we encounter challenges like that. They tend to complain to us, most of them are thieves in general.

***Questioner: What are the health challenges?***

**Officer G4:** there are other children coming from the street who are sick, for instance there is a child who came with diabetes but when we took him he was mischievous, that is, he has problems, we took him there but he beats the owner's children, he does not want to supervised, They are brutalized, they are raped, they are hurt, but if you tell him to go back home, he doesn’t agree, he wants to be treated and roam in the streets.

***Questioner: For you Social Welfare, have you ever encountered challenges when you take children to health services?***

**Officer G4:** When we take them to the health services, we are also part of the health service, so we are given approval whether treatment should be free or not, so we do not have a big challenge. The biggest challenge is for cases that go to court to get a doctor to write PF3 form for you, others tend to refuse to fill in those forms, but we get good cooperation.

**Officer G2:** Another challenge is when children are taken to the court, they give you guarantors, but we don't have a center for victims, you have to ask them to stay with someone or return to their own street, the law requires the local Chairman to make sure the child is there. The child escapes, the court demands you have to find him.

***Questioner: What are the opportunities available to help children who work on the streets, or what methods do you use to find or find those opportunities?***

**Response 1:** Organizations help us to provide insurance to those with difficult circumstances, so insurance helps them in health, they are also given capital to a certain amount, and we are being helped to reduce street children.

***Questioner: Are the available opportunities consistent with the needs of children who work on the streets and enable them to access health services?***

**Officer G2:** In a way, they are compatible, although not in all percentages, the children who receive health services, MOCSO provides insurance for children in homes and capital to reduce street children, especially those who go from dangerous places to safe places. We are a health department, we don't find it difficult at every health center or hospital. There is a welfare officer department. If there is a problem, we contact the welfare officer. The child is given an exemption, so when he leaves here, he goes to the hospital, he is given treatment.

***Questioner: How do you work with the community around you and do they make sure the children work and where do you get health and protection insurance?***

First, in the community around us we provide education and we call schools so as to talk to parents, the main source is in the family when parents have misunderstandings, we provide education in schools and we also encourage parents to join groups so that they can get rid of the issue of poverty and they can protect their children from sexual violence.
