# Supplementary material for: Challenges in accessing health care and socio-protection services among children living and working in streets in northwestern Tanzania: A qualitative study
Source: PLOS Glob Public Health. 2023 May 17;3(5):e0001916. doi: 10.1371/journal.pgph.0001916 (PMC10191300; doi:10.1371/journal.pgph.0001916)
Supplement: S1 Data — (ZIP) [file pgph.0001916.s001.zip › Data/CSO YY4.doc]

**CSO Y4**

**Mtoa jibu 1**: Railway children ni shirika ambalo sio la kiserikali na tunafanya kazi moja kwa moja na watoto wanaoishi mazingira hatarishi hususani watoto wanaoishi mitaani tuna ofisi zetu hapa Mwanza makao makuu ya shirika yako Dar es Salaam lakini pia tunatekeleza mradi wetu Dodoma tunashirikiana na mdau wetu shirika moja linaitwa TIZEBET hapo Dodoma ndio wao wana implement huo mradi ila sisi ndio tunawagawia fedha kuweza kuimplement kwa hiyo tunasema tupo Dar Es Salaam, Dodoma na Mwanza.

**Muulizaji**: Ningependa kujua majukumu yenu kama shirika la kijamii hapa jijini Mwanza?

**Mtoa jibu 1**: Kama nilivyosema awali tunafanya kazi na watoto wanaishio na kufanya kazi mitaani na shughuli tunazofanya sisi ni shughuli kubwa nne shugghuli ya kwanza ni **street outreach children** ambayo tunazunguka maeneo mbalimbali hapa jijini Mwanza ambako mtoto anapatikana katika wilaya ya Ilemela na Nyamagana. Katika wilaya ya Ilemela nyamagana yenye kata 39 kuna kata ambazo tunaziangalia ambapo watoto wengi wako hapo kuna kata ya nyegezi. Kata ya nyegezi ina stand ya mabasi kwa hiyo watoto wanaingia na kutoka tukishafika pale tunaongea nao. Watoto wengi wanaanza kuingilia hapo nyegezi na akishazoea ndo anaelekea maeneo mengine. Tumefanya kazi kata ya Nyegezi na pamba kwa muda mrefu, Natta, Mabatini na makaburi ya waislam maeneo ya pale kuna watoto wengi kwa sababu ni Karibu na stand ya mabasi watoto wanapatikana kwa wingi. Tunaangalia wilaya ya Nyamagana inachukua Kamanga na city center kwenye vivuko wakitokea Sengerema, Bukoba wanafikia pale Kamanga lakini wengine wanakuwa pale wanajua kuna ziwa hivyo kuogelea ni free kwa hiyo watoto wanakuwepo. Halafu tunaanglia kata ya Igogo. Igogo kwa hapa Mwanza ni kata fulani yenye matukio mbalimbali walevi. Pia kata ya Kirumba soko la mwaroni, Soko la kirumba sisi huwa tunafanya kazi na huwa tunaenda hayo maeneo na maeneo tunaenda na tunayatambua kupitia watoto wenyewe au wadau na serikali kwa hiyo huwa tunaenda pale. Kuna siku huwa tunaenda asubuhi sana, kuna siku ndani ya wiki tunaenda usiku na siku zingine tunaenda mchana na tunaenda kwa ajili ya kuwatambua watoto, kujenga uhusiano waweze kutuamini tuweze kufanya nao kazi. Lakini pia tunaenda pale tuweze kujua hali zao. Wakati mwingine unaenda pale unakuta mtoto anaumwa unamchukua unampeleka hospital. Pia wakati mwingine unamkuta mtoto ni mgeni amefika we need to rescue unamchukua wakati huo unafanya juhudi za kumrudisha kwao, wakati mwingine unaenda pale unawakuta wamelala kwenye mitaro wakati wa mvua inakuwa risk place. Pia tunafanya kazi na serikali inatusaidia maendeleo ambapo watoto wanalala au wanakaa na sisi tunaendelea kufanya nao kazi. Lakini pia kwenye hiyo street children outreach unakuta wanajamii wanatoa support wale mamantilie ama wasamaliawema tunawauliza watoto kule mtaani ni watu gani huwa wanawasaidia kwahiyo hao watu tunawatambua kwa kupitia watoto kwa namna mbali mbali na nyie mnawaamini mkiwa na shida unaenda kumwambia watoto wanatwambia.na sisi tunawaita community Championi. Sisi tunaenda kwa wale watu tunafanya nao mazungumuzo tunawatambua tunawapa training tunawashauri watoto wawapeleke Polisi kwa afisa ustawi wa jamii wanamandate ya watoto kuwatafutia makazi wengine hawafuati sheria. Wanawachukua wanaenda nao nyumbani ambayo ni hatari sana inatakiwa wawe na Barua ya Mwenyekiti ama polisi ili ikitokea tatizo inakuwa rahisi kutatua. Wakati mwingine unapigiwa simu unaambiwa huku stand mgambo wanawapiga watoto wa mtaani kwa hiyo sisi tunaenda pale tunaongea na polisi tunawachukua tunawapeleka kwa afisa ustawi wenyewe wanawatafutia vituo vya kukaa. Kuna familia nyingine huwa zinaweza kukaa na hao watoto wakati familia zao zinatafutwa zikipatikana tunampeleka mtoto. Kwa hiyo maximum ya mtoto kukuaa kwa mtu ni miezi 6, baada ya miezi 6 unarudi kwa afisa ustawi kumwambia kwamba nikaa nae nimepata details kwa hiyo wao ndo street person na afisa ustawi watajua njia nyingine ya kufanya kwa hiyo ndizo kazi tunazifanya. Halafu mtaani wengine wakati wa usiku tunaenda polisi lakini polisi hawajaleta desk lakini polisi wanajua watoto wanaishi wapi kwa hiyo mkienda na polisi inakuwa rahisi kwa ajili ya usalama wetu. Kwa sababu watoto wapokatika maeneo yanaweza kuhatarisha staff member wetu na polisi huyo anavaa kiraia akiwa na dhana zake lakini ili watoto wakaogopa na wasikimbie. Intervention ya pili nyingine tunayofanyan na inabeba Railway children na moyo upo na akili zetu zipo wa shirika yetu ipo inaitwa **family intergration** au watu wengine wanapenda kuita **family rule reification** ila inaitwa family intergration ambayo ni process unaweza kufanya reification (reification ni kumpata motto na kumrudisha nyumbani kwao). Tuna watoto hapa Mwanza majority wanatokea mikoa mingine na wachache wanatoka hapa Mwanza. Mwingine amekuja kwa sababu amefiwa na wazazi, mwingine amekuja kwao ni masikini, mwingine ameambiwa kuna samaki mkubwa anatema maji anakuja kumwamgalia na wengine sababu ya peer pressure, kwa hiyo solution haiwez kuwa moja kutokana na sababu zilizomleta huku mtaani. Zamani tulikuwa tukijua mtoto ametokea kigoma tunamchukua tunamrudisha kigoma, hii ikaleta changamoto ya time consuming hivyo watoto walikuwa hawatoi taarifa kamili tukawa tunafanya tracing by networking Tukaamua staff atakuwa anaondoka na mtoto mara familia yake inapokuwa imepatikana kwa hiyo mtoto tukishamhoji ndo tunaanza kutafuta familia, zile details anazotoa ndo tunazitumia kupata familia yake. Kwa kujua jina la shule, ama siri zote za maeneo ya kwao kwa hiyo tunampigia simu Afisa ustawi wa kule ambapo mtoto alipokuwa anaishi, Mwenyekiti ama Mtendaji mpaka tunapata familia ya mtoto. Tukishapata familia ya Yule mtoto tunaenda kufanya household assessment kujua hali ya afya, uchumi wa familia hali ya mahusiano na maswal aya ukatili wa kijinsia natunatrace maswala ya afya baada ya hapo tunaanza kufanyia hizo assessement ili kujua the basic needs za huyo mtoto je mtoto akirudi hatowza rudi mtaaani. The are some family Unakuta mtoto anaishi na bibi na babu Yule mtoto anakwambia madam tunalala njaa kama kuna uwezekano wa kuwapa mtaji wafanye biashara ili kuinua kipato hapo inakuwa ni uongo maaana hao wazee hawataweza kufanya biashara tunatoa children support another support ya kuangalia nani ataweza kukaa na huyo mtoto kwa hiyo familia. Familia amabazo wanajiweza tunatoa business support kama mitaji na tunawapa startup Fulani ya kufanya biashara waweze provide needs za familia.pia tunashirikiana na afisa maendeleo wa maeneo husika waweze if possible waweza kuwepo kwenye hiyo startup program. We provide CHF insuarance ambayo tunaamini after one year a family inaweza kupay hiyo insuarance. Kwahiyo inategemea na mahitaji ya familia, na sio kwamba tunaweza kuprovide each and everthing huwa tunaongea na wadau au serikali wanatoa vifaa ila sio kwa kiasi kikubwa wakati mwingine mashirika yanatusaidia.watoto wengi wanaokuja mitaaniwana mawili either ameandikishwa akadrop shule unakuta motto yupo katika umri wa shule sisi tunajitahidi kumuandisha kwa wale wanaohitaji na sio wote. Na baada ya ratification ya kugundua kuwa mazingira yapo salama na hakuna risk na wazazi wapo tayari kumpokea na kumlea motto yupo tayar kurudi nyumbn ikifika muda anafanyiwa verifiykurudi nyumban ikiwa ndani au nje ya mwanza kwa some of the cases anaondoka na afisa stawi akiwa motto wa kike anaondoka na afis ustawi au afisa ustawi wa eneo analo kwenda anapelekwa Tunapokwenda kumkabithi uongozi wa mtaa serikali ya kijiji au afisa mtendaji lazima awepo, afisa maendeleo representative wa shule ambapo motto atasoma majirani na sisi lazima tuwepo tunamkabithi motto kazi iliyobaki ni kufanya followup baadae motto akiendelea vizuri tunagraduate case tunaendelea na watoto wengine hiyo ndo kazi tunafanya kwenye family work. Kwenye family work kuna kitu kinaitwa parenting, wototo wengi huja mitaani kwasababu ya kukosa malezi kutoka kwa familia zao auukosefu wa malezi na mafunzo kwa wazazi kwa hiyo mtoto akifika katka umri wa adolescent anaanza kuonyesha some behavior mzazi anaona solution kumtandika fimbo nyingi kumbe hajui kuwa motto yupo katika umri wa adolescent kwenye umri huu motto anatakiwa kusaidiwa hivi na hivi kwa hiyotunaenda kwenye jamii tunafanya identification gaps kuwa hawa watoto wamekuja mtaani na hawa watakuja mtaani kwa sababu hii tunakutana na care giver na familia kwenye groups kwenye maeneo yao tunatoa elimu inatwa parental child development child care education ya week kumi na moja tunafanya na guide kupitia child development kumuelekeza mzazi, care giver jinsi ya treating the adolescent child. Pia tunafanya kazi na vijana wa mtaani ambao kipaumbele chao sio kurudi majumbani wanapenda kuishi maisha yao binafsi na wanajikuta wanajiingiza na risk behavior kwa hiyo tunakutana nao kwenye makundi yao kwenye maeneo ambapo wanapatikana tunaongea nao tunawawezesha wanatulia tunawapa ushauri ili waweze kujitegemea na kuwa transform tabia zao kabla ya kuwawezesha na wapate awareness and kutulia. We connect them to vocation training center or other training centers so that they get training and knowledge on how to depend on themselves and not engage on street illegal activities lastly we make prevention on street children previously we were responding on the problem but now we have gone extra miles we make measures. We prepare community dialogs preparing session with the society with our facilities like speaker to the society as organization to educate them on concerning on child right abuse,child right, how to report cases concerning any abuses cases concerning child’s needs jinsi ya kupunguza idadi ya watoto walioko mtaani na kuepukana na kokaine na madawa mengine ya kulevya kwa hiyo tunawaita wadau, Mwenyekiti, Mtendaji na polisi ili waweze kutusaidia kuwapeleka kwa Afisa ustawi wa jamii. Kwa hiyo tunashirikiana na wadau kuandaa redio program, kwa ufupi sana hizo ndo shughuli tunazozifanya.

**Muulizaji**: Nyinyi mnafanya vipi kuwasaidia hawa watoto ambao wanapitia kwenye mazingira hatarishi?

**Madam Irene**: sisi tunawarescue hao watoto, Tunawapatia msaada wa matibabu, endapo motto amefanyiwa ukatili tunatoa aids pia tunawalinda endapo wakikamatwa na kupelekwa mahakamani sisi tunajitahidi haki za motto zinazingatiwa. Pia tunawashikamanisha na familia zao hawa watoto, hizo ni baadhi ya shughuli tunazozifanya kwenye shirika.

**Muulizaji**: Je ni watoto wangapi wanaoishi mitaani mnawasaidia katika kupata huduma za afya

**Mtoa jibu 1**: hatuna clear number ya namnana tunavyowasaidia kuwapatia huduma za afya mana hawa vijana ni mali ya serikali kwa hiyo akienda hospital bila Fedha hawawezi kumpatia matibabu otherwise mpaka apewe document za kutoka Ustawi wa Jamii. Sisi tumekubaliana kila kina wa mtaani akiugua tunamchukua tunampeleka Hospital ya Kanisa la makongoro AIC maeneo ya misheni tunapewa dawa tunamchukua mtoto tunampeleka kwa mama ntilie tunampatia dawa kwa ajili ya usimamizi wa dose. Tunatumia exemption Ikitokea mtoto amebakwa tunamchukua tunampeleka Hope child center ama Seokotuore ni rahisi kwa sabubu bei ni poa anatibiwa tunaenda pale wale wanamtibu kwa interseption. Tunaprovide NHIF card kwa watoto sita ambao watoto wamekaa kwa muda mrefu mtaani.

**Muulizaji**: Je kuna changamoto ya huduma ya afya kwa upande wa PF3 kwa mtoto aliyejeruhiwa kwa hospital huwa mnafanyaje?

**Mtoa jibu 1**: sisi kwa sababu tunajua wadau hatupati changamoto tunamchukua mtoto tunampeleka Hospital The charity ambayo inatumia exemption inampatia matibabu kwa urahisi.

**Muulizaji**: Na katika hayo makundi wanaoishi mitaani katika mazingira hatarishi je idadi ya watoto wa kike wanakuwemo?

**Mtoa jibu 1**: Idadi ya watoto wa kike ni ndogo sana kulinganisha na wa kiume. Wa kike sio rahisi sana kuwa watoto wa mtaani sana sana mtoto wa kike ni markertable mtaani atapata kazi kwa mama ntilie kuosha vyombo ama kazi za ndani ama (house girl). Nisiseme hawapo wapo lakini idadi yao ni ndogo sana kulinganisha na wakiume.

**Muulizaji**: Je ni misaada ipi mnaitoa kama shirika kuwasiadia watoto wanaoishi mitaani kuwalinda dhidi ya ukatili wa kiafya?

**Mtoa jibu 1**: Tunatoa elimu kwanza kwa watoto dhidi elimu dhidi ya ukatili. Huwa tunawashauri mara nyingi usilale maeneo flani peke yako, usilale sehemu yenye giza, usipokee vitu kutoka kwa strangers. Hakuna mtoto ambae yuko chini yetu huwa anaenda mwenyewe huwa tunampeleka manake unakuta wengine hawawezi kujieleza lazima tuwapeleke tukatoe maelezo zaidi. Tunawaeleza kutoa taarifa za ukatili kwa watu wanaowaamini kama social workers. Tunatoa elimu kwa adolescent children kuhusu elimu ya edolescence na sexual reproductions. Tunatoa training and test on HIV/AIDS kwa watoto.

**Muulizaji**: Ni vikwazo vipi hasa ambavyo mnakutana navyo kama shirika mnapokuwa mkitoa msaada watoto wanaoishi na kufanya kazi mtaani?

**Mtoa jibu 1**: Negative perception Serikali inaaimni kwamba sisi railway children tunapomkuta mtoto wanaamini wewe una promote waendelee kuishi mtaani kwa kuwa mtoto akiugua unampeleka hospital kwa hiyo hicho ndicho kikwazo tunachokutana nacho. Cards za nhif tulizowakatia zilikuwa haizifunction matibabu mengine kama x-ray, operations tukaingia gharama nyingine ili mtoto apate huduma ya afya. Pia tunapopata kesi za ukatili wa kijinsia ni ngumu sana kupata watuhumu. Mtoto anapobakwa tunamchukua tunampeleka hospital anatibiwa lakini watuhumu hawamhitaji kwa sababu anakuwa hakumbuki nini amefanyiwa. Kuhusu huduma ya afya ni ngumu sana watoto wa mitaani kwenda hospital akapewa msaada ni ngumu sana yaani ngumu sana mtoto wa mtaani akienda kwa mfano makongoro pale kupokelewa ni mtihani kwa sababu watoto wa mtaani wana changamoto nyingi. Changamoto nyingine ukienda hospital unaambiwa mara X-Ray ni mbovu. Changamoto nyingine jamii huwa haiamini watoto wa mtaani hata ukienda kutoa perception bado wengine wanakuwa wanafanyiwa ukatili na wanapewa majina machafu, wakati mwingine wanapigwa na mgambo kwa sababu ya perception ya watoto wanaoishi mtaani. Changamoto nyingine jamii inawachukulia watoto wa mitaani ni tofuti na watoto wengine inashindwa kutuelewa Railway children hawafanyi kazi kwa sababu ukitoa hawa wanakuja hawa kwa sababu mauaji yanaendelea wale watoto wanajikuta ni watoto wa mitaani. Tabia ya watoto ni cyclic wanatoka hawawanakuja hawa mtaani.

**Muulizaji**: Ni namna gani mnafanya kazi na idara za afya pamoja na vituo vya afya kama shirika na kutoa huduma kwa watoto waishiona na kufanya kazi mtaani na kuwalindwa na ukatili wa kijamii?

**Mtoa jibu 1**: Tuna vitambulisho vyetu vya watoto wa mtaani na hospital inakuwa inatambua vitambulisho vyetu kwa hiyo mtoto akienda na hicho kitambulisho lazima atapata matibabu. Kwa hospital zinazotoa misaada zaidi ni seko toure manake pale kuna Afisa ustawi wa jamii kwa hiyo tunapata taarifa masaa 24 kwa hiyo kimbilio lolote ni pale seko toure. Pale hatujawhi kwenda tukashindwa kupata matibabu kwa sababu exemption nyingi za vikao vyetu wanazielewa lakini pia mfano Hopital za Butimba. Nyamagana Makongoro na Buzuruga tukienda pale Tunakutana na Afisa ustawi wa jamii anatu link tunaenda tunapata matibabu. Lakini sisi pia kwa kutambua swala la ukatili tunatoa training ya madaftari kwenye vituo vya afya inapopatikana kesi tuweze kupata PF3 na kesi mahakamani tunahakiksha tunajaza madaftari. Tunaona kabisa alikuwepo mwendesha mashitaka wa serikali aliweza pia kusaidia. Lakini pia tuliweza kufanya vikao pamoja na inter perception tunatoa guideline kipi kifanyike na yeye ana sort out ndivyo huwa tunafanya. Kwa mfano kijana madawa ya kulevya wanakuja madakitari for training kutoka Bugando ikitokea tatizo tunakuwa sambamba na hospital hizo.

**Muulizaji**: Ni kwa namna gani mnafanya kazi na jamii inayowazunguka watoto wa mitaani wanaofanya kazi na kulindwa dhidi ya ukatili?

**Mtoa jibu 1**: Kuelimisha jamii namna ya kusaidia watoto kukuonyesha kutoa ripoti chanya polisi na afisa ustawi wa jamii to connect us with hospital center ndo hizo huduma tunatoa, Pia tunwaelimisha kuna bima za afya ambazo tumeziboresha kwa watu 6 akiwemo baba ambapo itawasaidia kupata matibabu kwa wakati.

**Muulizaji**: Ni fursa zipi ambazo mnatoa katika kuwasaidia watoto waishio na kufanya kazi mitaani kwa kupata huduma za afya na kuwalinda na ukatili wa kijinsia katika jamii?

**Mtoa jibu 1**: Fursa zipo nyingi kwa serikali ya mtaa na Afisa Ustawi wa jamii anaweza kutoa kibari kupata matibabu bure katika hospital kwa wahanga wote wa ukatili wa kijinsia kwa hiyo wanaweza wakaenda wakatumia hizo fursa. Fursa zingine wanaweza kwenda kupimwa vipimo maternity na vipimo vingine bure kupitia msaada wa serikali ama mashirika. Hizo ndo fursa ambazo wanatumia kupata huduma ya matibabu bure.

**Muulizaji**: Je mnatumia mbinu gani kupata hizo fursa?

**Mtoa jibu 1**: kuwa Karibu sana na DMO tunashare title, kwamba kuna siku ya ukimwi ,nyota ya kijani kwa hiyo ananimbia niwambie watoto pia kupitia Afisa ustawi wa jamii anashare na sisi taarifa kwamba huduma ya afya watakuwa watoa huduma sehemu flani kwa hiyo wapeleke watoto. Au wale maafisa ustawi ambao wapo kwenye vituo vya afya mara nyingi huwa wanatupatia taarifa kuna fursa mbali mbali pale hospital wanaweza kuzifnya au wakati mwingine watendaji wanatupa taarifa kwamba kwenye kata yetu wiki hii tutakuwa na chanjo ya watoto umri huu na huu kwa hiyo mnaweza kuwaleta watoto wenu wapatiwe chanjo. Ama mnaenda nao wanachanjwa mnarudi, Kwa mfano wakati wa COVID19 watoto walikuwa wanapatiwa sanitizer, barakoa, sabuni. Wakati mwingine watendaji wanakwambia wiki hii watagawa vifaa kwa hiyo unawapeleka wanapatiwa hayo mahitaji.

**Muulizaji**: Na mlikuwa mnatumia mbinu gani kuwambia hao watoto mana wako scattered?

**Mtoa jibu 1**: Kwa wiki tunakutana nao mara 2. Mnawambia wiki hii kuna nini wiki ile kuna nini. Kuna kinga ya kinywa na meno tafadhari tunaomba mwende kuangalia meno yako kama mazima ama lah! Na hakikisha unawapa taarifa watoto wengine kwa hiyo ni mbinu mojawapo ambayo tunaweza kutumia. Mbinu nyingine tunawambia mama ntilie kwamba watoto wakija waambie waende uwanjani kuna huduma inatolewa bure. Mara chache sana hasa kwa watoto wa nyegezi stand huwa tunamwambia Yule mtoa matangazo atangaze watoto wote waende sehemu flani. Ama Yule mtoa fursa unamwambia akifika sehemu flani atangaze watoto waende sehemu flani. Wakija kwenye semina tunawaelimisha.

**Muulizaji**: Je hizo fursa zinazotoka ni kweli zinaendana na mahitaji yao?

**Mtoa jibu 1**: wakati mwingine unakuta watoto ni tofauti, fursa tofauti, watoto wa mtaani wana magonjwa ya ngozi, mlipuko wa magomjwa wanapigana, wasichangiane damu vingine vinakuwa out kabisa.
