# Supplementary material for: Challenges in accessing health care and socio-protection services among children living and working in streets in northwestern Tanzania: A qualitative study
Source: PLOS Glob Public Health. 2023 May 17;3(5):e0001916. doi: 10.1371/journal.pgph.0001916 (PMC10191300; doi:10.1371/journal.pgph.0001916)
Supplement: S1 Data — (ZIP) [file pgph.0001916.s001.zip › Data/WATOTO WA MITAANI.doc]

Mahojiano kati G4 mkila afisa ustawi wilaya ya nyamagana na G2 muhanda afisa ustawi na jamii mwanza jiji.

**Muulizaj**i: je ni njia zipi hasa mnazitumia katika kuhakikisha watoto wanaoishi na kufanya kazi mtaani na wanapataje huduma za kiafya na kulindwa dhidi ya ukatili wa kijinsia na kijamii.

**Afisa G4**: kwanza kabisa mchana hatupendi kukaa kwa hiyo hatuwezi kusema kuna huduma tunawapa watoto wa mitaani ila kuna wale watoto wanaokuja wametumwa na wazazi wao kuuza karanga, ndizi ambao wanazunguka mtaani jioni wanarudi nyumbani hasa wakati wa likizo kwa hiyo hao hata wakiugua wana familia, Anafanya kazi mtaani jioni anarudi nyumbani kwao lakini sababu za kiafya ana familia yao lakini kuna wale ambao wanatoka mkoa mwingine wanakuja wanaamua kulala tunatakiwa tumtambue ili tuweze kumrudisha nyumbani, tunawauliza kwa nini umekuja mtaani wanajibu kwamba nimekuja kuomba pesa, ama nimekuja kuomba pesa nipate vifaa vya shule kwa hiyo ule mwanzo tunapenda arudi nyumbani kwao hatupendi akae mtaani kwa sababu mtaani kuna changamoto mtaani huwezi kusema utampa huduma kwanza utampata wapi leo kalala hapa kesho kalala sehemu nyingine.

**Afisa G2**: kwa kuongezea pia kuna wale watoto ambao wanaingia mtaani wanaokolewa na Maafisa Ustawi au mashirika yanayodili na watoto wa mitaani kwa hiyo watoto kama hao family sheet zinapofahamika makwao watoto wale wanapokuwa wanarudishwa kwao wanapitishwa kwanza kwenye vituo vya Afya kwenda kuchekiwa afya zao baada ya hapo tunaenda kuwaunganisha na familia zao.

**Muulizaji**: Je kuna mashirika ya kijamii ambayo yanafanya kazi ya kuwasaidia watoto wanaoishi na kufanya kazi mitaani katika kuwawezesha kupata huduma za Afya?

**Afisa G4**: Ndio kuna mashirika mengi mojawapo shirika kubwa ni railwa**y Chidren** ambao asilimia kubwa ndio wanafanya kazi na watoto wa mitaani na wakiumia wanawapeleka Hospital lakini pia kuna kituo cha **Hospital ya CF** ilikuwa inawahudumia watoto wenyewe pia walikuwa wanajua lakini pia tuna **Cheka Sana** na yenyewe wakiumia watoto wanawapa msaada wote lakini ni pamoja na kuwapa msaada wa kuwarudisha kwao.

**Muulizaji**: Kwa sasa ni mashirika mangapi yaliyoko wilayani kwako yanajihusisha na kusaidia watoto wanaoishia na kufanya kazi mitaani?

**Afisa G4**: yapo matatu lakini kuna mengine mawili madogo madogo ambayo ni Farijika, Fanisi, Rafiki kwa watoto na Railway children, cheka sana.

**Muulizaji**: Dodoso Je katika mashirika haya ni mangapi yana nguvu ya kuwasidia watoto hawa mitaani?

**Afisa G4**: Mashirika yote

**Muulizaji**: Ni nini hasa majukumu yenu hususani katika mashirika yanayohusika na kusaidia watoto wa mitaani?

**Afisa G4**: kwanza hawa watoto hata mashirika wakiyapata ni lazima tuwape ushauri kwa nini wapo mtaani, Pia lazima tuwasiliane na Afisa Ustawi wenzetu kwa mfano mtoto akisema ametokea Wilaya ya Ngara lazima Tuwasiliane na Afisa Ustawi wa Ngara ili atusaidie kufanya ufatiliaji wa kujua familia je kule ina hali gani ni jukumu la Afisa ustawi kujua kwa nini mtoto amekuja huku na kule kuna tatizo gani lakini kujua Afya ya mtoto kuna fomu tunatakiwa tuzijaze ili tujue Afya ya mtoto anaendeleaje tukishamaliza hata kama kuwarudisha sisi ndio tunatoa kibali, kwanza tunatoa kibali cha watoto hao kukaa kwenye makao na pia tunatoa kibali cha watoto hao kurudi kwao.

**Afisa G2**: Tunapowarudisha wale watoto hao ni wajibu wa Afisa Ustawi kuendelea kutoa Elimu kwa wazazi juu ya swala zima la watoto kuja kufanya kazi tunaendelea kuwaelimisha kuhusu swala zima kumpeleka motto sehemu ya kufanya kazi nje ya matakwa yake kama kufanya kazi za ndani au udangulo, kutoa elimu kwa swala la malezi ili vitendo hivyo visiendelee.

**Muulizaji**: Je mashirika haya yanajihusisha na watoto wanaoishi na kufanya kazi mitaani inatakiwa kufanya nini hasa katika kuwasaidia watoto hawa?

**Afisa G4**: Mara nyingi tuna wale social worker unajua watoto wako hivi ukiongea kwa ukali na wenyewe watakudanganya kwa hiyo mashirika yanafanya urafiki na wale watoto na kuwa na social worker ambae wanamuita teacher kwa hiyo unaweza kukutana na mtoto mmoja ukaongea nae hata mwezi mzima mpaka mtoto anawakubali na anaamini na anaweza kufunguka lakini mtoto ukimkuta siku ya kwanza anaweza asifunguke ama amechoka mitaani anaona apana nimekosea nirudi lakini ukimkuta Yule mtoto amekaa sana mtaani inatakiwa hasa urafiki sasa yale mashirika yana wafanyakazi ambao kila siku wako mtaani wanakuwa Karibu na wale watoto lakini pia kuwaandaa na kuwaleta kwetu kwa ajili ya kupata hayo mahitaji mengine ambayo tunakuwa tumesaidiwa. Pia kuna shirika lingine linaitwa Upendo Daima wenyewe wana kituo chao wanakaa nao watoto, wakati mwingine unakuta wanawachukua wanaenda kukaa nao unakuta watoto wenyewe wamekaa mtaani sana wameona hali ni ngumu sanaa wanataka kusoma basi wanaenda kukaa nao. Hayo hayo mashirika wanawapatia vifaa vya shule lakini pia kuongea nao kuhakikisha wanatoa fedha kuwawezesha.

**Afisa G2**: wakati mwingine wanasaidia wazazi wa wale watoto ambao walikuja huku,watoto wanaondoka kwenye familia zao kipato chao kinakuwa kigumu wanawapa fedha kwa ajili ya mtaji ili wainue kipato chao wakati mwingine watoto wanatoka majumbani kwao kwa sababu ya kipato.

**Muulizaji**: Je ni misaada ipi mnaitoa katika mashirika haya ya kijamii kuwasidia watoto wanaoishi na kufanya kazi za mitaani kupata huduma za afya na kuwalinda dhidi ya ukatili wa kijinsia jamii

**Afisa G4**: kwenye mashirika sisi tunafanya nao kazi pamoja, lakini pia wakati mwingine mtoto anaweza kuumia mashirika hayana fedha sisi tunatoa msamaha wanaenda kwenye Hospital zetu wanatibiwa kwa msamaha tunahakikisha wamepata msamaha na wamepata matibabu. Tunafanya nao kazi bega kwa bega wakituletea tunafanya kwa sababu hawawezi kufanya kazi bila kutushirikisha.

**Afisa G4**: Hata zile fomu za matibabu zinatoka Ustawi wa jamii.

**Muulizaji**: Je ni vikwazo vipi hasa mnakutana navyo kutoka kwenye mashirika ambayo yanahusika kutoa msaada kwa watoto wanaoishi na kufanya kazi mitaani?

**Afisa G4**: Vikwazo sio vingi kwa sababu wao wanatusaidia bila wao sisi wenyewe tusingeweza.wanatusaidia kutafuta watoto hakuna vikwazo vikubwa tunaishi vizuri.Changamoto zipo kwa watoto wa mitaani sio kwa mashirika ni kwa watoto wenyewe hawatoi ushirikiano hata hao unaowaona mitaani wamekaa wanaenjoy wameshapita kwenye mashirika mbalimbali tunawapeleka tunaongea na wazazi lakini baada ya miezi wanarudi na mnakutana nao mitaani na wanakuita Madam wanakusalimia anakufahamu mahari flani mlikutana kama anakukejeli hivi hizo ni changamoto za watoto.

**Afisa G4**: Changamoto nyingine tunayokumbana nayo watoto tunaojihusisha nao huwa hawatoi taarifa kamili kwa sababu labda kule kwao alitoka ameiba au amefanya kitu kibaya sasa hiyo inatusumbua akienda kule anaeleza kingine akija huku pia anaeleza kingine kwa hiyo inatusumbua.

**Afisa G4**: Changamoto nyingine tena huwa tunawachukua tunawapeleka kwa mtu anayeaminika akae nae ili ajiandae kwenda nyumbani kwao wanawaibia wale watu wetu wengine wanaobaki kitu kikubwa ama Fedha akaondoka ama mabegi ya nguo kwa hiyo tunakutana na changamoto za namna hiyo. Wanakuja kutulalamikia nimeibiwa begi, Afisa nimeibiwa wengi ni wezi kwa ujumla kabisa.

**Muulizaji**: Je changamoto za kiafya

**Afisa G4**: kuna watoto wengine wanakuja mtaani wanaumwa, kuna mtoto alikuja ana changamoto ya sukari lakini tulipompeleka nako ni mkorofi yaani ana matatizo tunampeleka kule lakini anapiga watoto wa mwenye nyumba hataki wamsimamie pia naye anatushitua. Wanapata ukatili wanabakwa pia wanalawitiwa wanaumizwa lakini ukimwambia kurudi nyumbani hakubali yaani anataka atibiwe harafu aendelee kudunda mtaani.

**Muulizaji**: Kwa nyinyi Ustawi wa Jamii mlishawahi kupata changamoto mnapokuwa mnawapeleka watoto kwenye huduma za kiafya?

**Afisa G4**: Tunapowapeleka kule kwenye huduma za kiafya sisi ni sehemu ya huduma ya afya kwa hiyo sisi ndio tunapewa kibali atibiwe bure au atozwe kwa hiyo hatuna changamoto kubwa . changamoto kubwa ni kwa kesi zinazoenda mahakamani kupata dakitari wa kukuandikia PF3 wengine wanakuwa wanakataa kujaza hizo form lakini tunapata ushirikiano vizuri.

**Afisa G2**: Changamoto nyingine watoto wanapopelekwa mahakama inakupa wadhamini manake hatuna kituo cha waharifu inabidi uombe akakae kwa mtu ama arudi mtaani manake sheria inamtaka Mwenyekiti wa Mtaa wa pale ahakikishe mtoto yupo. Mtoto anatoroka mahakama inakudai unampata wapi.

**Muulizaji**: Je ni fursa zipi zilizopo kuwasaidia watoto wanaofanya kazi mitaani, au mnatumia mbinu gani kutafuta ama kupata hizo fursa?

**Mtoa jibu 1**: Mashirika yanatusaidia kutoa bima kwa wale wenye mazingira magumu kwa hiyo bima zinawasaidia kwenye afya pia wanapewa mitaji kwa kiasi flani tunakuwa tunasaidiwa kupunguza watoto wa mitaani.

**Muulizaji**: Je fursa zinazopatikana zinaendana na mahitaji ya watoto wanofanya kazi mitaani na kuwawezesha kupata huduma za afya?

**Afisa G2**: kwa namna flani zinaendana ingawa si kwa asilimia zote wale watoto wanopata huduma za afya MOCSO wanatoa bima na kwa watoto waliopo majumbani na mitaji ili kupunguza watoto wa mitaani hususani wale ambao wanatoka sehemu hatarishi kwenda sehemu salama. Sisi ni idara ya afya huwa hatupati ugumu kila kituo cha afya ama Hospital Kuna idara ya Afisa ustawi endapo inapotokea tatizo tunawasiliana na Afisa ustawi mtoto anapewa msamaha kwa hiyo akitoka hapa kwetu anaenda Hospital anapewa matibabu hatujawahi kukosa huduma.

**Muulizaji**: Je ni kwa namna gani mnafanya kazi na jamii inayowazunguka na wanahakikisha watoto wanafanya kazi na mnapata wapi bima za afya na ulinzi?

**Afisa G4**: kwanza jamii inayotuzunguka tunatoa elimu na tunaita mashuleni tunazungumza na wazazi, chanzo kikubwa ni kwenye familia wazazi wanapogombana tatizo linaanzia hapo, tunatoa elimu tunapita mashuleni na pia tunajaribu kuongea na wazazi na kujiunga kwenye vikundi ili waweze kujikwamua katika swala la umaskini ili waweze kuwalinda watoto wao na ukatili wa kijinsia kwa watoto.

Nawashukuru.
