# Supplementary material for: Challenges in accessing health care and socio-protection services among children living and working in streets in northwestern Tanzania: A qualitative study
Source: PLOS Glob Public Health. 2023 May 17;3(5):e0001916. doi: 10.1371/journal.pgph.0001916 (PMC10191300; doi:10.1371/journal.pgph.0001916)
Supplement: Attachment — Submitted filename: Response to Reviewers_PGPH-D-23-00312.docx [file pgph.0001916.s002.docx]

Journal Requirements:

**Reviewer #1**

Line 27: Delete "to" between among & CLWS.

**Response:**

"to" has been deleted as suggested. see page.2, line 27
Line 52: Write UN in its long form (United Nations) and then put the abbreviation UN in brackets.

**Response:**

UN has been written in long form and its abbreviation in brackets. See page.3, line 53
 Line 59: ADD "is" between It and estimated & ADD "are" between "up and to".

**Response:**

“is" has been added between It and estimated & "are" has been added between "up and to" see page.3, line 60
 Line 64: Write HIV/AIDS in long form first and then put the abbreviation in brackets

**Response:**

HIV/AIDS has been written in long form first and its abbreviation in brackets. See page 4, line 65.
 Line 74: ADD "families” after polygamous and a semicolon before "also".

**Response:**

This sentence has been omitted from the paragraph. See page 4
 Line 76: ADD "in" between conducted and Tanzania.

**Response:**

“In” has been added between conducted and Tanzania. See page 4, line 78
 Line 112: DELETE comma in the abbreviation CL,WS so that it appears as CLWS.

**Response:**

The comma has been deleted and the word now reads as CLWS. See page.6, line 114
 Line 120: DELETE "s" from the word “examines” so that the word will read examine & EDIT the CSO’s to be CSOs

**Response:**

"s" has been deleted and the word now reads as examine. See page.6, line 122
 Line 134: Authors need to consider deleting one among these two words (used employed) since they mean the same here.

**Response:**

The word used has been deleted remaining with employed. See page.7, line135
 Line 138: I suggest to authors to ADD "to have" between reported & around 39.6%

**Response:**

"to have" has been added between reported & around 39.6%. see page.7, line 139

 Line 142: I suggest to authors to REPLACE "cities" with “areas”.

**Response:**

“areas” has been used instead of cities. See page.7, line 143
 Line 143: I suggest to authors to consider adding "which" between facilities and include OR change "include" to be including.

**Response:**

"which" has been added between facilities and includes. See page.7, line 144
 Line 148: ADD "of" between "provision & health".

**Response:**

"of" has been added between "provision & health". see page.7, line 150.
 Line 163: DELETE “Key Informants Interviews” and use only its abbreviation.

**Response:**

“Key Informants Interviews” has been replaced by its abbreviation. See page.8, line 164.
 Line 167: DELETE “Focus Group Discussion” and use only its abbreviation

**Response:**

“Focus Group Discussion” has been replaced by its abbreviation. See page.8, line 168.

 Line 479: DELETE Civil Society Organizations” and use only its abbreviation without brackets.

**Response:**

Civil Society Organizations has been replaced by its abbreviation. See page.23, line 483.
 Line 500: DELETE comma after South so that it will read “South Africa”.

**Response:**

The comma after South has been deleted and now reads as “South Africa”. see page.23, line 504.
 Line 508: CORRECT the spellings for Pakistani by DELETING “I” so that it will read Pakistan.

**Response:**

The “I” has been deleted and the word now reads as Pakistan. See page 24, line 512.
 Line 531-533: The sentences (((The provision of medicines however is not based on medical laboratory diagnostic reports. However, monitoring of dosing has always been a challenge.)))) I suggest to authors to consider merging the two sentences by adding a semicolon instead of full stop after reports and DELETE "However", so that the sentence will read as follows: ""The provision of medicines however is not based on medical laboratory diagnostic reports; and monitoring of dosing has always been a challenge."""

**Response:**

“However,” has been deleted and the sentence now reads “The provision of medicines however is not based on medical laboratory diagnostic reports; and monitoring of dosing has always been a challenge”. see page.25, line 536.
 Line 542: CHANGE “drives” to be “drivers”.

**Response:**

“drives” has been changed to “drivers”. see page.25, line 546.
 Line 543: The word “Consequences” to be a lower case (i.e., to start with small “c”.

**Response:**

The word consequences now begin with a lower case. See page 25, line 547.
 Line 577: DELETE comma after South so that it will read “South Africa”.

**Response:**

comma after South has been deleted and it now reads as South Africa. See page26, line 581.
 Line 613: DELETE "s" in individuals so that it will read individual CSOs

**Response:**

"s" has been deleted and the word now reads as individual. See page 28, line 617.
 Line 652: BACKSPACE "s" so that “clinic s” will read "clinics"

**Response:**

"s" in “clinic s” has been backspaced and now reads as "clinics". see page.30. line 656.
 Line 658: REPLACE “Civil society organization” with its abbreviation CSOs

**Response:**

“Civil society organization” has been replaced with its abbreviation “CSO”. see page.30, line 662.
 In Fig 1. Conceptual Framework on Health Care and Socio-Protection services among CLWS as summarized from different studies elsewhere:
I suggest to the authors to make corrections in Fig. 1 in the following areas
o On the DRIVERS OF CLWS: --Hunder (Food shortage) NEEDS to be corrected to "Hunger (Food shortage) &
o On "Enhancing Access to Health Care and socio-protection services" - the "Local Governmental" SHOULD BE EDITED TO BE "Local Government"

**Response:**

The spelling mistake has been corrected to Hunger…See Fig. 1

“Local Governmental” has been changed to “Local Government” as suggested.

**Reviewer #2**

"Challenges in accessing health care and socio-protection services among children living and working in streets in northwestern Tanzania: A qualitative study"
reveals important insight on health economic considerations for Tanzania's childhood socio-protection.
It is further applicable to the wider African and Global South context.
Yet in order to make these findings truly transnationally comparable evidence base cited should be diversified and expanded much beyond wealthy OECD countries - related finding.
This refers particularly to the developing LMICs health systems across the Global South particularly those that quite comparable to Tanzania and leading Emerging Markets.
For this purpose I warmly recommend to consider for inclusion a few of the below listed sources alongside with additional ones at authors own disposal:

[https://globalizationandhealth.biomedcentral.com/articles/10.1186/s12992-020-00590-3](https://globalizationandhealth.biomedcentral.com/articles/10.1186/s12992-020-00590-3#inbox/_blank)
[https://www.mdpi.com/1660-4601/16/17/3043](https://www.mdpi.com/1660-4601/16/17/3043#inbox/_blank)
[https://bmcmedicine.biomedcentral.com/articles/10.1186/s12916-022-02639-z](https://bmcmedicine.biomedcentral.com/articles/10.1186/s12916-022-02639-z#inbox/_blank)
[https://www.tandfonline.com/doi/full/10.1080/13696998.2023.2178164](https://www.tandfonline.com/doi/full/10.1080/13696998.2023.2178164#inbox/_blank)
[https://www.mdpi.com/2071-1050/15/2/1372](https://www.mdpi.com/2071-1050/15/2/1372#inbox/_blank)
[erepository.uonbi.ac.ke/bitstream/handle/11295/161245/Mihajlo%20Jakovljevic.pdf?sequence=1](http://erepository.uonbi.ac.ke/bitstream/handle/11295/161245/Mihajlo%20Jakovljevic.pdf?sequence=1#inbox/_blank)
[https://www.tandfonline.com/doi/full/10.1080/13696998.2022.2160608](https://www.tandfonline.com/doi/full/10.1080/13696998.2022.2160608#inbox/_blank)
[https://resource-allocation.biomedcentral.com/articles/10.1186/s12962-022-00405-9](https://resource-allocation.biomedcentral.com/articles/10.1186/s12962-022-00405-9#inbox/_blank)
[https://www.frontiersin.org/articles/10.3389/fpubh.2022.1062425/full?trk=public_post_feed-article-content](https://www.frontiersin.org/articles/10.3389/fpubh.2022.1062425/full?trk=public_post_feed-article-content#inbox/_blank)
[https://www.frontiersin.org/articles/10.3389/fpubh.2022.793648/full](https://www.frontiersin.org/articles/10.3389/fpubh.2022.793648/full#inbox/_blank)
[https://www.hindawi.com/journals/bmri/2022/5514793/](https://www.hindawi.com/journals/bmri/2022/5514793/#inbox/_blank)
[https://health-policy-systems.biomedcentral.com/articles/10.1186/s12961-022-00822-5](https://health-policy-systems.biomedcentral.com/articles/10.1186/s12961-022-00822-5#inbox/_blank)
[https://pubmed.ncbi.nlm.nih.gov/30568938/](https://pubmed.ncbi.nlm.nih.gov/30568938/#inbox/_blank)
[https://www.ncbi.nlm.nih.gov/pmc/articles/PMC9647898/](https://www.ncbi.nlm.nih.gov/pmc/articles/PMC9647898/#inbox/_blank)
[https://www.tandfonline.com/doi/full/10.2147/TCRM.S307587](https://www.tandfonline.com/doi/full/10.2147/TCRM.S307587#inbox/_blank)
[https://pubmed.ncbi.nlm.nih.gov/34676266/](https://pubmed.ncbi.nlm.nih.gov/34676266/#inbox/_blank)
[https://www.ncbi.nlm.nih.gov/pmc/articles/PMC9644335/](https://www.ncbi.nlm.nih.gov/pmc/articles/PMC9644335/#inbox/_blank)
[https://www.ncbi.nlm.nih.gov/pmc/articles/PMC9397501/](https://www.ncbi.nlm.nih.gov/pmc/articles/PMC9397501/#inbox/_blank)
I remain willing to review the revised manuscript version.

**Response:**

Two articles with relevance to this study have been adopted and cited to help in emphasizing on the desired message. See. Page.4, line 85; reference 10 & 11.

**Reviewer #3**

Abstract:
Line 27: “defiling” is not the right word here.

**Response:**

This has been changes to enhancing. See page.2, line 27.
Line 36: what does “donor dependency” mean?

**Response:**

This sentence has been rephrased and now reads s “who depend on donations from public passersby”. see page.2, line 36.
The introduction:

Lines 66-75: The introduction is lengthy, so I suggest removing this part that will render the intro more focused on CLWS in Tanzania.

**Response:**

This paragraph has been omitted. see page.4
Methods:
Line: 134: a reference to the phenomenological approach is needed.

**Response:**

a reference to the phenomenological approach has been added. See page.7, line 135, reference 21.

Line 145: references that explain systematic random, purposive, and convenience sampling are needed.

**Response:**

references that explain systematic random, purposive, and convenience sampling have been added, see page. 7, line 146, 148 and 152. references 22, 23 and 24.
Line 172: A reference for thematic content analysis is missing.

**Response:**

A reference for thematic content analysis has been added, see page.8, line 173, references 25.
Line 180: a reference that describes the importance of investigator triangulation is needed.

**Response:**

A reference that describes the importance of investigator triangulation has been added, see page.8, line 181, reference 27.
Line 182: an in-text citation for NVivo 12 is missing.

**Response:**

An in-text citation for NVivo 12 has been added, see page.9, line 184. reference 28.
Results:
216: The authors mentioned that five themes emerged but discussed only four themes and one subtheme.

**Response:**

This has been corrected and now reads as Four key themes and one sub theme, see page 11, line 217.
The results section is too long. I recommend that the authors cut some of the non-essential verbatim of the included quotes.

**Response:**

Non-essential verbatim of the included quotes have been deducted to make the results section shorter but still meaningful, see page.10, line 212, page.11, line 219-220, page.12, line 238-39, 242, 244-45, 249 & 252-54, page.13, line 265-66 & 285, page.14, line 287-89 % 298-300, page.16, line 335, 355 &357. page.17, line 365-66.

**Reviewer #4**

A lot of typo and grammatical errors.

**Response:**

All grammatical errors and typos have been amended throughout the document.

Title needs to provide a more comprehensive overview of the study's objective and focus.

**Response:**

The revised version of the manuscript provides contents that suit the current title and objectives.

Objective is not clear, needs to be rephrased.
**Response:**

The objective has been revised, now it reads, “ To explore the role of CSOs, preventing barriers and existing opportunities in enhancing the access to health care and socio-protection services among CLWS in Mwanza city, northwestern Tanzania.” See Abstract Section Line 26-28 page 1
